# Recent Advances and Future Directions in Sonodynamic Therapy for Cancer Treatment

**DOI:** 10.34133/bmef.0080

**Published:** 2024-12-27

**Authors:** Priyankan Datta, Sreejesh Moolayadukkam, Dhrubajyoti Chowdhury, Adnan Rayes, Nan Sook Lee, Rakesh P. Sahu, Qifa Zhou, Ishwar K. Puri

**Affiliations:** ^1^ Department of Aerospace and Mechanical Engineering, University of Southern California, Los Angeles, CA 90089, USA.; ^2^ Iovine and Young Academy, University of Southern California, Los Angeles, CA 90089, USA.; ^3^ Mork Family Department of Chemical Engineering and Material Science, University of Southern California, Los Angeles, CA 90089, USA.; ^4^ Alfred E. Mann Department of Biomedical Engineering, University of Southern California, Los Angeles, CA 90089, USA.; ^5^ Department of Materials Science and Engineering, McMaster University, Hamilton, ON L8S 4L8, Canada.; ^6^ School of Biomedical Engineering, McMaster University, Hamilton, ON L8S 4L8, Canada.

## Abstract

Deep-tissue solid cancer treatment has a poor prognosis, resulting in a very low 5-year patient survival rate. The primary challenges facing solid tumor therapies are accessibility, incomplete surgical removal of tumor tissue, the resistance of the hypoxic and heterogeneous tumor microenvironment to chemotherapy and radiation, and suffering caused by off-target toxicities. Here, sonodynamic therapy (SDT) is an evolving therapeutic approach that uses low-intensity ultrasound to target deep-tissue solid tumors. The ability of ultrasound to deliver energy safely and precisely into small deep-tissue (>10 cm) volumes makes SDT more effective than conventional photodynamic therapy. While SDT is currently in phase 1/2 clinical trials for glioblastoma multiforme, its use for other solid cancer treatments, such as breast, pancreatic, liver, and prostate cancer, is still in the preclinical stage, with further investigation required to improve its therapeutic efficacy. This review, therefore, focuses on recent advances in SDT cancer treatments. We describe the interaction between ultrasound and sonosensitizer molecules and the associated energy transfer mechanism to malignant cells, which plays a central role in SDT-mediated cell death. Different sensitizers used in clinical and preclinical trials of various cancer treatments are listed, and the critical ultrasound parameters for SDT are reviewed. We also discuss approaches to improve the efficacies of these sonosensitizers, the role of the 3-dimensional spheroid in vitro investigations, ultrasound-controlled CAR-T cell and SDT-based multimodal therapy, and machine learning for sonosensitizer optimization, which could facilitate clinical translation of SDT.

## Introduction

Despite advances in deep-tissue solid tumor treatment based on conventional approaches, such as surgical intervention followed by chemotherapy and radiation therapy, patient outcomes remain poor. The primary reasons for the low prognosis are the heterogeneous tumor microenvironment (TME) supported by an abnormal vasculature, higher interstitial fluid pressure, and, most importantly, the adaptation of cancer cells that results in their acquisition of resistance against therapy [1–3]. Additionally, the “cold” solid TME prevents immune cell activation, which further limits the efficiency of the immunotherapy-based cancer treatment [4].

In this context, sonodynamic therapy (SDT) is an emerging noninvasive, targeted cancer treatment. SDT typically uses low-intensity pulsed ultrasound (LIPU) in the ranges of 0.5 to 5 W/cm^2^ and 0.035 to 3 MHz for intensity and frequency, respectively [5–7]. This allows deep-tissue penetration, target specificity, and opening of the blood–brain barrier (BBB) [8,9]. Thus, SDT has the potential to overcome the current conventional therapeutic challenges for solid tumor treatment.

The primary components of SDT are the sonosensitizers. They are generally tumor cell-selective drugs that can be activated by either ultrasound or light, and are nontoxic and chemically stable [10,11]. The underlying mechanism of sonosensitizer activation with ultrasound is a complex phenomenon that is yet poorly understood. It is reported that sonosensitizer activation in response to the ultrasound is similar to that of conventional photosensitizers in photodynamic therapy (PDT).

The most acceptable theory is that sonosensitizers accumulate selectively within tumor cells after administration due to the higher metabolic rates of these cells. Subsequent application of ultrasound facilitates energy transfer as sound energy is transformed into light. The sensitizers absorb this released photon energy, moving from the ground to an excited state. During relaxation from the excited state, sonosensitizers produce reactive oxygen species (ROS), particularly cytotoxic singlet oxygen, which triggers tumor cell death [8,10,12–15].

A recent phase 0 clinical trial for high-grade glioma and preclinical investigations reported direct evidence of oxidative stress generation following SDT. This supports the assumption of a sonosensitizer activation pathway and ROS-mediated tumor cell death in a manner similar to PDT [6,11,16–20]. Further, the low-frequency ultrasound used for SDT allows it to penetrate to greater depths (>10 cm) than PDT (<1 cm) [8] and is, therefore, expected to be more effective for solid tumor treatment.

Clinical trials of SDT have only been reported for brain tumors. For other cancer treatments, such as breast cancer [21,22], pancreatic cancer [23–25], and prostate cancer [26,27], SDT remains in a preclinical stage. Most preclinical in vitro investigations rely on 2-dimensional (2D) monolayer cultures, but 2D investigations fail to mimic the physiological conditions of in vivo tumors, such as the physical barrier imposed by the extracellular matrix and the supporting stromal cells, and the concentration gradient of oxygen in the tumor.

Therefore, future preclinical in vitro studies should investigate the influence of SDT on 3D cellular structures (such as multicellular spheroids), which represent the in vivo physical barrier, and oxygen and nutrient concentration variations more closely than a 2D monolayer culture [28]. These 3D cell structures also mimic sonosensitizer diffusion and thus cellular uptake more closely as occurs in tumors under in vivo SDT conditions. Based on this rationale, the use of 3D spheroids can help bridge the gap between current preclinical in vitro experiments and animal models. The simultaneous use of different types of sonosensitizers with a 3D model to evaluate the most effective sonosensitizer combinations can further enhance SDT efficacy. However, to the best of our knowledge, the literature in this context is sparse, and we have introduced the discussion here for the first time.

In this review, we (a) systematically discuss the underlying mechanisms of SDT, (b) describe different sonosensitizer classes used for various cancer treatments along with optimal ultrasound parameters for preclinical in vitro and in vivo experiments, and discuss (c) recent advances in SDT for different types of cancer treatments and (d) current status of clinical trials for brain tumors. Finally, a roadmap is presented for (a) clinical translation that highlights the key aspects required to develop more effective sonosensitizers, (b) investigation of combined SDT and ultrasound-controlled chimeric antigen receptor (CAR)-T therapy with a high-throughput 3D spheroid platform, and (c) incorporation of machine learning techniques for screening optimal sensitizer combinations.

## SDT Mechanisms

### Sonochemical pathway

**Fig. 1.**
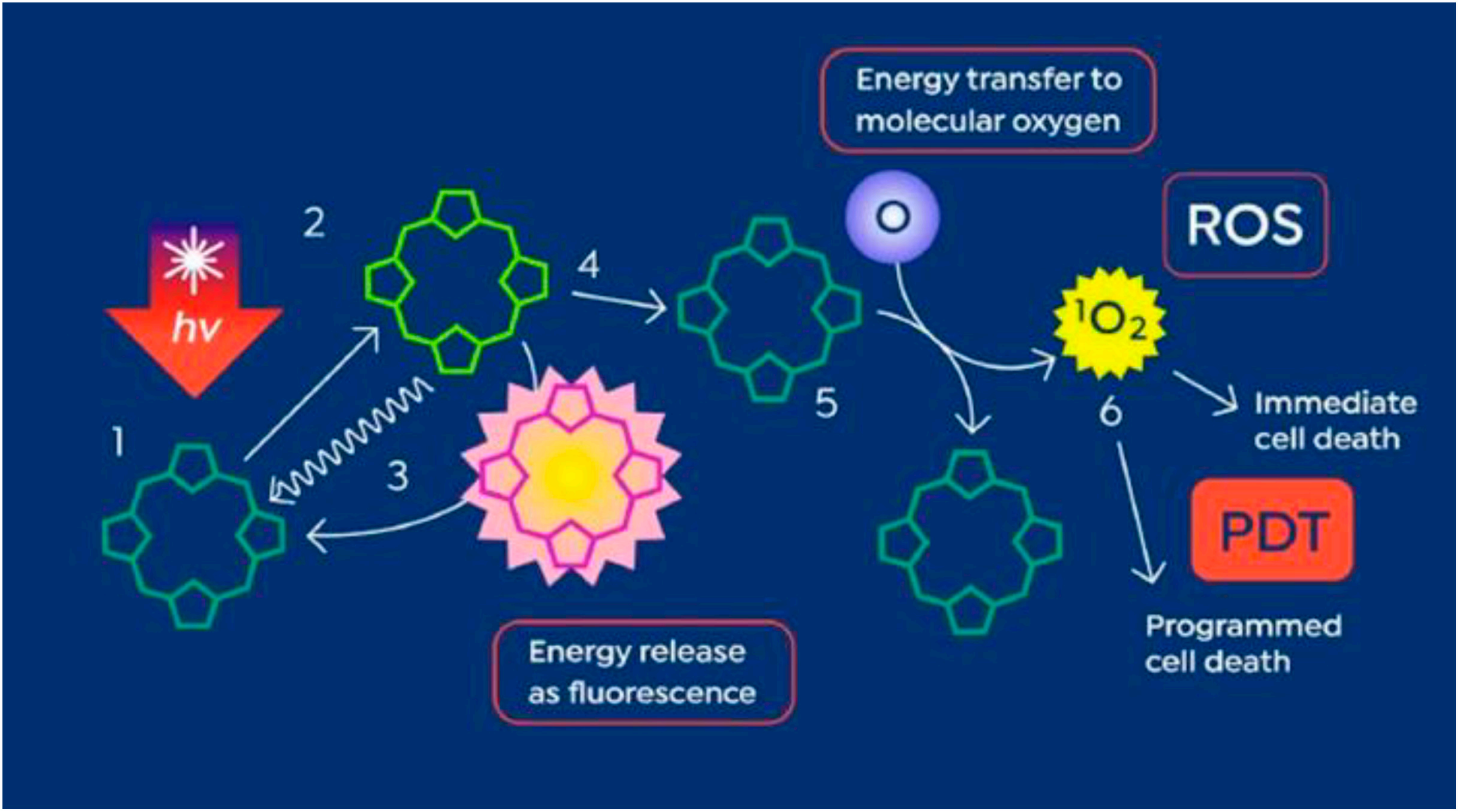
Schematic of a photosensitizer activated by light followed by photodynamic reaction-mediated tumor cell death. This image is reproduced with permission from [11]. https://creativecommons.org/licenses/by/4.0/ PpIX is a photosensitizer that absorbs light and becomes excited (steps 1 and 2). Deexcitation (steps 3 and 4) releases fluorescence energy at a higher wavelength than of the incident light. The released energy produces singlet oxygen (steps 5 and 6), which produces ROS and triggers the cell death pathway.

The interaction between ultrasound and a sonosensitizer and, thereby, the associated energy transfer is still unclear [18,29]. The most acceptable theory for the onset of antitumor activity through SDT is similar to that for PDT, where a photoactive molecule, or photosensitizer, absorbs energy from light and participates in photodynamic reactions (Fig. 1). For SDT, sonosensitizers are organic or synthetic molecules that are activated by the acoustic energy of the applied ultrasound [8]. This activation pathway is complex, for which the most acceptable hypothesis is sonoluminescence [30].

#### Sonoluminescence

**Fig. 2.**
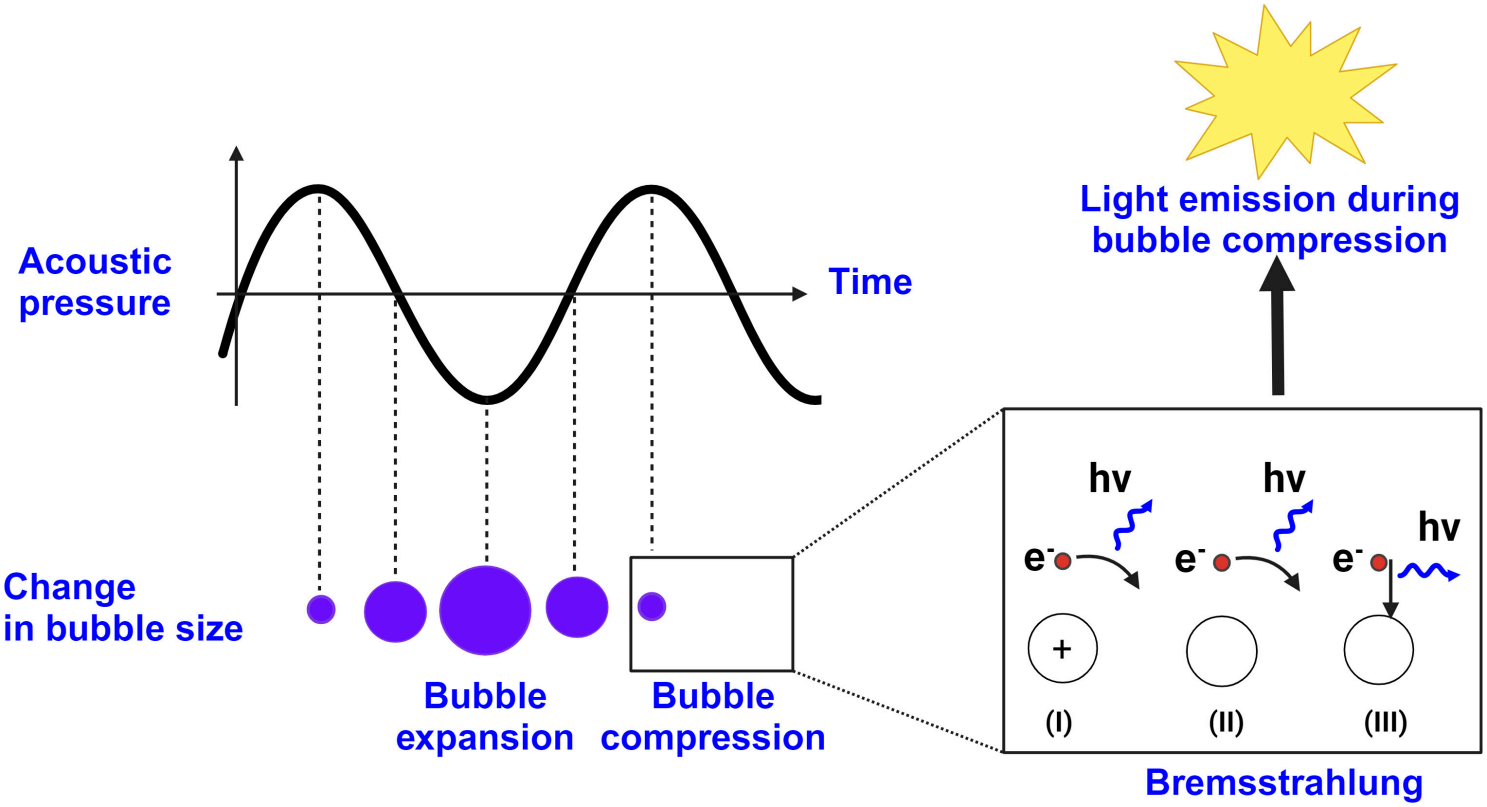
Schematic illustration of sonoluminescence onset during single microbubble compression when the microbubble is subjected to ultrasound. The microbubble starts to grow during the negative cycle of the applied ultrasound and reaches its maximum. The bubble size reduces as the ultrasound field reverses toward its minima. At its minimum size, the temperature rise inside the bubble is sufficient to cause weak gas ionization, producing plasma consisting of electrons and ions. The deceleration of electrons near (I) positive ions, (II) atoms, or (III) their recombination produces electromagnetic radiation, known as “Bremsstrahlung”. This electromagnetic radiation in the visible range is called sonoluminescence.

Sonoluminescence occurs when either an endogenous gas bubble, also known as a vacuole, near the tumor tissue or an exogenously administrated microbubble collapses, leading to visible light generation [30,31]. The acceptable hypothesis for sonoluminescence onset is thermal bremsstrahlung [30], which occurs because of bubble gas compression during the positive cycle of the applied ultrasound, producing a large temperature rise (10,000 to 20,000K) that results in gas ionization. The ionization produces sonoluminescence, i.e., when free electrons decelerate near positive ions or atoms, resulting in continuous visible light emission [31–36]. Figure 2 presents a schematic of the sonoluminescence onset during microbubble collapse induced with ultrasound.

#### Sonosensitizer activation pathways

**Fig. 3.**
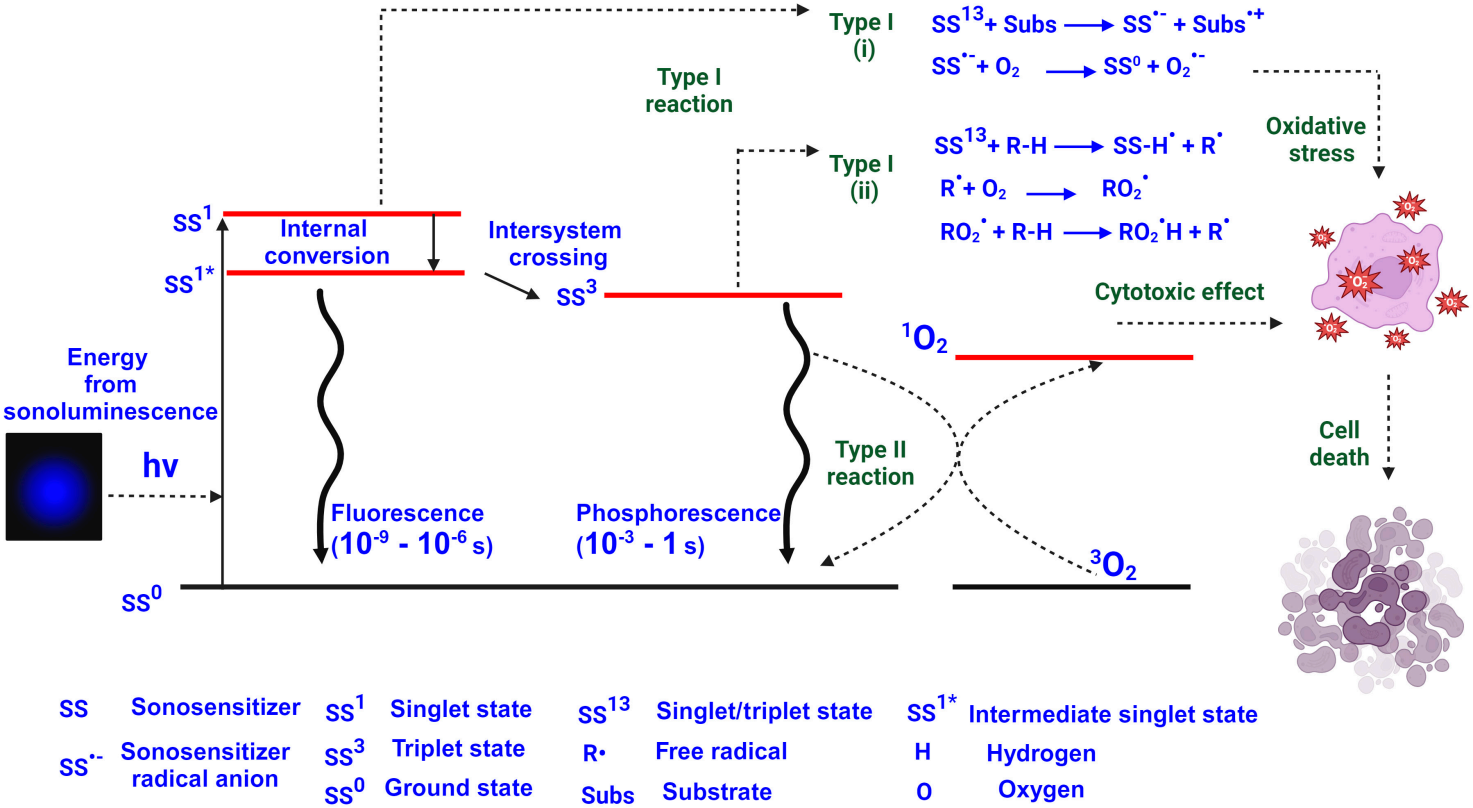
Schematic illustration of sonosensitizer (SS) activation upon receiving energy from the sonoluminescence of a microbubble. SS is excited to its singlet excited state (SS^1^) from a ground state (SS^0^). SS^1^ deexcites either directly to SS^0^ or through SS^3^ to SS^0^. During deexcitation, either one or both type I and type II reactions occur. During a type I reaction, free radicals are generated either from a singlet or triplet excited state of the sonosensitizer. For a type II reaction, only a triplet excited state of the sonosensitizer reacts with molecular oxygen, producing singlet oxygen. Free radicals or singlet oxygen produces a cytotoxic effect on tumor cells, resulting in cell death.

The working principle of a sonosensitizer is similar to that of a photosensitizer [10,37–39]. A sonosensitizer absorbs energy from emitted light during a sonoluminescence event (Fig. 3). Through this energy absorption, a sonosensitizer electron is excited from its ground state to a higher energy molecular orbital, resulting in a short-lived sonosensitizer excited state (or singlet state, SS^1^). During deexcitation, the sonosensitizer undergoes internal conversion to an intermediate singlet state, SS^1^*, followed by fast decay during 10^−6^ to 10^−9^ s to its ground state, SS^0^, conserving electron spin multiplicity. Another possible pathway for sonosensitizer deexcitation is through intersystem crossing (ISC), first from its excited singlet state to an excited triplet state, SS^3^, by means of spin inversion and then to its ground state. This pathway is a spin-forbidden process where spin conservation is not maintained; hence, it involves a longer decay time to the ground state (10^−3^ to 1 s) [37].

**Fig. 4.**
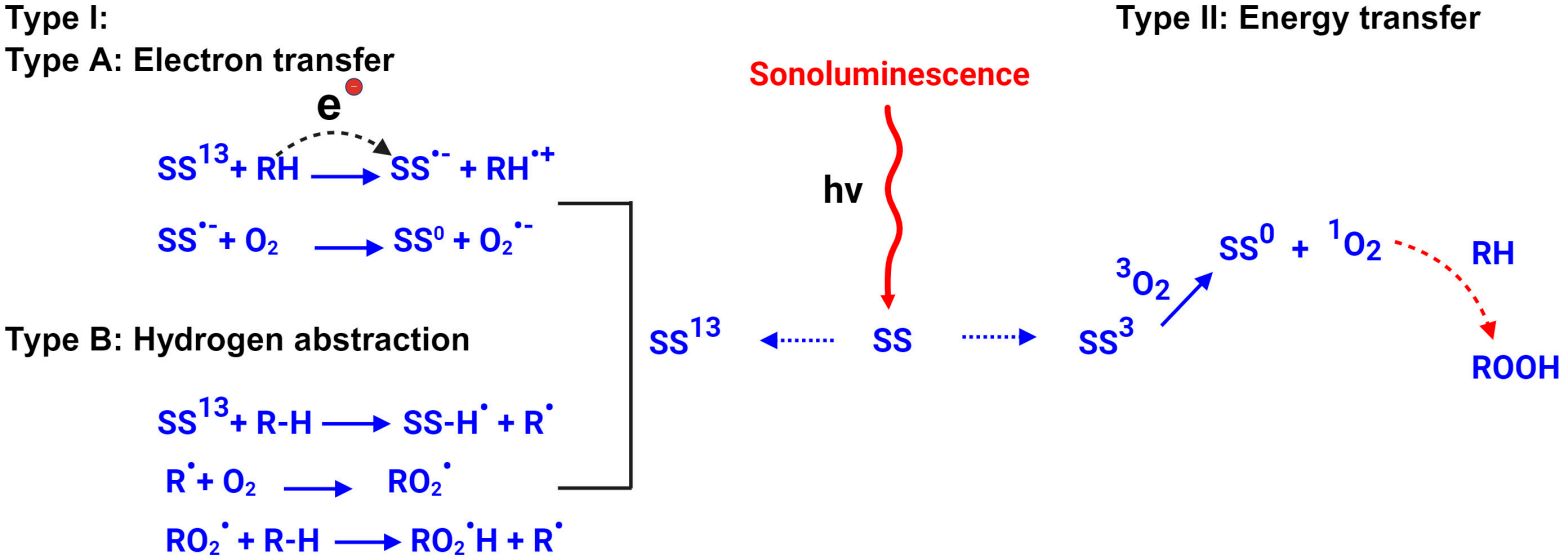
Schematic representation of the 2 types of reaction paths after sonosensitizer activation [37]. The type I reaction involves either a singlet or triplet excited state of the sonosensitizer, where electron or hydrogen atom transfer produces intermediate, short-lived free radicals (or ROS). A type II reaction is a direct energy transfer process where the triplet excited state of the sonosensitizer reacts with the ground state molecular oxygen, producing highly reactive, cytotoxic singlet oxygen (^1^O_2_), which participates in different cell damage pathways.

During deexcitation, the sonosensitizer can induce 2 types of reactions, type I or type II (Fig. 4). Type I reactions involve either the singlet or triplet excited state of the SS^13^ and can be further classified into type A and type B subtypes. In subtype A, the sonosensitizer is reduced from the singlet excited state and forms radical anions (*SS*^·−^) upon gaining an electron from a substrate that interacts immediately with oxygen to generate highly reactive short-lived oxygen radicals ($O_{2}^{\cdot -}$). For subtype B, the excited sonosensitizer state is reduced by hydrogen atom transfer, generating intermediate reactive free radicals. These free radicals react with molecular oxygen and generate ROS. The ROS from type A or B trigger cytotoxic pathways, such as cell membrane lipid peroxidation and cell death [37].

Type II reactions only involve the triplet state of the sonosensitizer and interact directly with molecular oxygen at its ground state, ^3^O_2_. This interaction results in a spin inversion and generates cytotoxic singlet oxygen, ^1^O_2_, at 2 different energy states depending on the occupancy of the electrons, either in the same orbital or in different orbitals. Different orbital occupancy leads to a higher energy state for singlet oxygen ($\Sigma_{g}O^{1}\sim 37\;\text{kCal}$) that has a shorter lifetime, less than 0.33 ms [37]. The same orbital occupancy yields a low-energy state of singlet oxygen ($\Delta_{g}O^{1}\sim 22\;\text{kCal}$) with a longer lifetime that induces cytotoxic reaction pathways and cell death [37].

#### Pyrolysis

The primary trigger for tumor cell death with SDT is through ROS, which can also be produced by acoustic cavitation-induced pyrolysis [8]. During a complete ultrasound cycle, a microbubble, either an endogenous microbubble or exogenously administrated, grows to a maximum size, following which its size reduces to a minimum volume during a duration that depends on the intensity of the applied ultrasound [40–43]. The bubble gains potential energy during its expansion, where the maximum potential energy gained by the bubble is based on its maximum radius. As the bubble compresses, its stored energy is transformed into mechanical energy through the motion of the bubble–liquid interface and into chemical, heat, and light energy [44,45].

Being several thousand kelvin, the temperature rise during bubble compression [46] is sufficient to cause free radical (OH·, H·) generation due to the pyrolysis of water near the gas–liquid interface [30]. About 10^−4^ times the maximum potential energy, roughly ~10^10^ eV for a maximum bubble radius of 30 μm, is required to form these free radicals [45]. Once produced, the free radicals can escape through the gas–liquid interface, producing further cytotoxic damage.

**Fig. 5.**
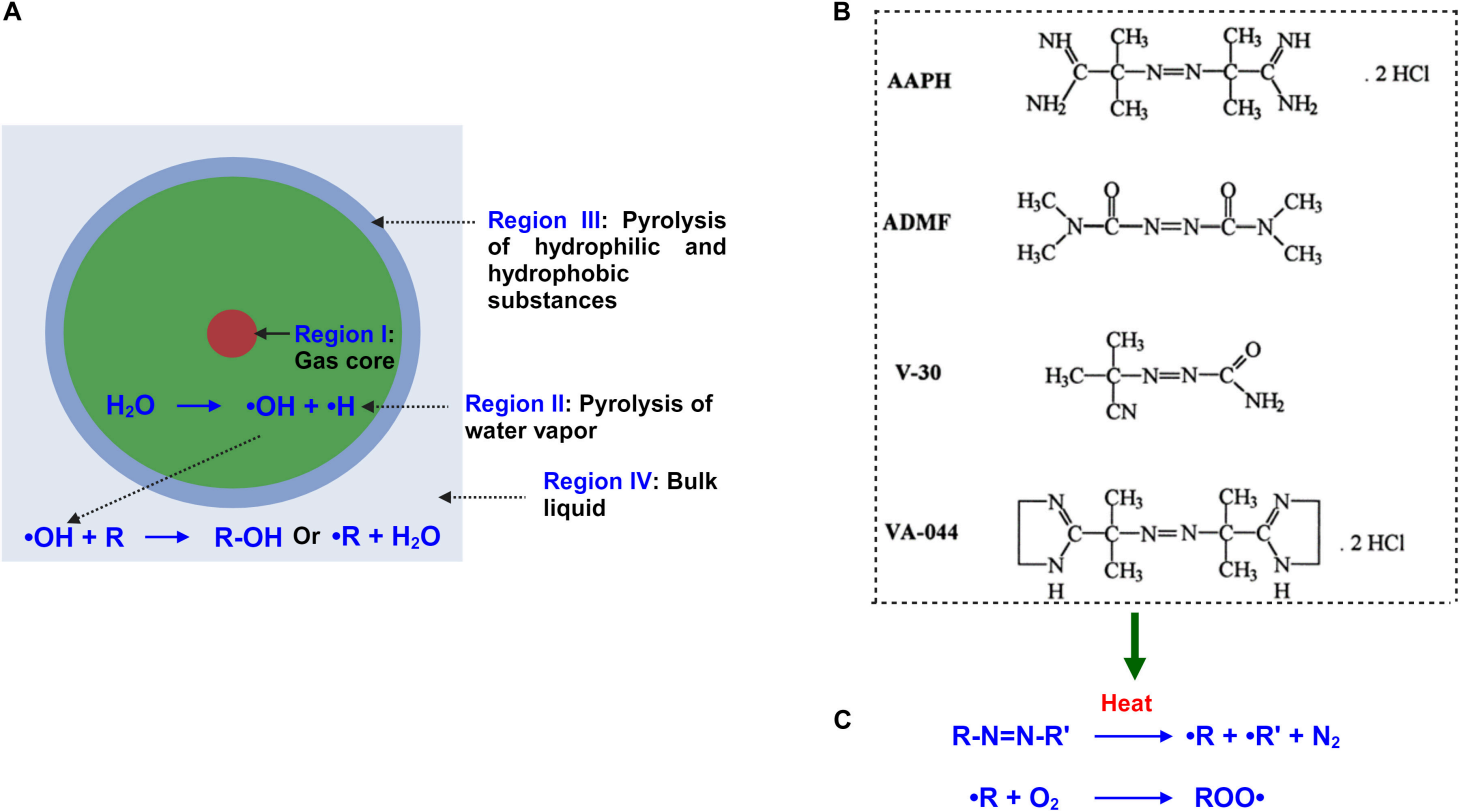
(A) Schematic representation of the sonochemical process during the acoustic cavitation of a microbubble. Region I: Maximum temperature rise zone due to gas compression inside the bubble during the positive cycle of the ultrasound. Region II: Pyrolysis of water vapor molecules forms highly reactive free radicals (OH·, H·). Region III: Pyrolysis of hydrophilic and hydrophobic substances. Region IV: Bulk liquid zone, where free radicals escape and react with surrounding organic molecules. (B) Different water-soluble azocompounds can generate free radicals through pyrolysis and can serve as sensitizers in SDT. AAPH, 2,2′-azobis(2-methylpropionamidine) dihydrochloride; ADMF, 1,1′-azobis (*N*,*N*′-dimethylformamide); VA-044, 2,2′-azobis (*N*,*N*′-dimethyleneisobutyramidine) dihydrochloride (VA-044); V-30, 2-(carbamoylazo)-isobutyronitrile. This image is reproduced with permission from [12], Copyright 2006, John Wiley and Sons. (C) Schematic representation of the generalized pyrolysis reaction of an azo compound-based sensitizer and generation of peroxyl radical (ROO∙) in the presence of oxygen.

A schematic diagram of the sonochemical processes occurring when a microbubble undergoes acoustic cavitation is shown in Fig. 5A. The cytotoxic damage caused by extracellular free radicals that are generated due to acoustic cavitation of the microbubble alone is debatable since these radicals are highly reactive with a very short half-life, e.g., 1 ns for OH∙, and have short diffusion distances, e.g., 5 nm for OH· [8]. The half-lives for different types of free radicals that can be generated by SDT are summarized in Table 1.

**Table 1.** A summary of different types of ROS that are generated during SDT [47]

Type	Half-life at 37 °C	Properties
Singlet oxygen (^1^O_2_)	1 μs	Strong oxidant
Superoxide anion (${O_{2}}^{-}\cdot$)	1 μs	Good reductant, poor oxidant
Hydroxyl radical (OH·)	1 ns	Extremely reactive, low diffusion distance; might not be directly responsible for the biological effect
Peroxyl radical (ROO·)	10 ms	Lower oxidizing ability compared to hydroxyl radicals but has a high diffusion
Alkoxyl radical (RO·)	1 μs	Intermediate reactivity between hydroxyl and peroxyl radicals

In addition to the pyrolysis of water vapor, the pyrolysis of the sonosensitizer itself and the generation of other intermediate, low reactive free radicals, such as peroxyl radicals, have been hypothesized [8] to cause cytotoxicity during SDT. Experimental observation of azocompounds used as sensitizers confirms that sonosensitizer pyrolysis with low-frequency ultrasound (50 kHz) application produces fewer reactive peroxyl radicals with a higher diffusion range and half-life [12]. Different types of water-soluble azocompounds and their chemical structures that can produce free radicals during pyrolysis are shown in Fig. 5B and C.

### Sonomechanical pathway

#### Effect on mechano-sensitive cation channel (Piezo 1) opening

The mechanical force exerted during SDT due to ultrasound application can trigger the opening of calcium ion channels such as Piezo 1 [48] at the plasma membrane of the tumor cells. This causes Ca^+2^ ion influx from the extracellular space within the cytosol [48]. Further release of Ca^+2^ ions from the endoplasmic reticulum within the cytosol ultimately leads to Ca^+2^ concentration overload. Under normal physiological conditions, Ca^+2^ intake by mitochondria is low due to the lower permeability of the inner mitochondrial membrane (IMM) [49,50]. The overload condition allows the mitochondrial permeability transition pore (mPTP) complex to open the IMM to balance the Ca^+2^ concentration within the cytosol [50,51]. However, the high influx of Ca^+2^ may result in osmotic swelling followed by IMM rupture.

Mitochondrial membrane rupture causes further release of cytochrome c from the mitochondria within the cytosol and thereby triggers the programmed cell death pathway or apoptosis [51–53]. Another possibility is that the higher Ca^+2^ influx can disrupt the electron transport chain and, consequently, increase the mitochondrial ROS generation rate due to the leakage of the electrons [51].

**Fig. 6.**
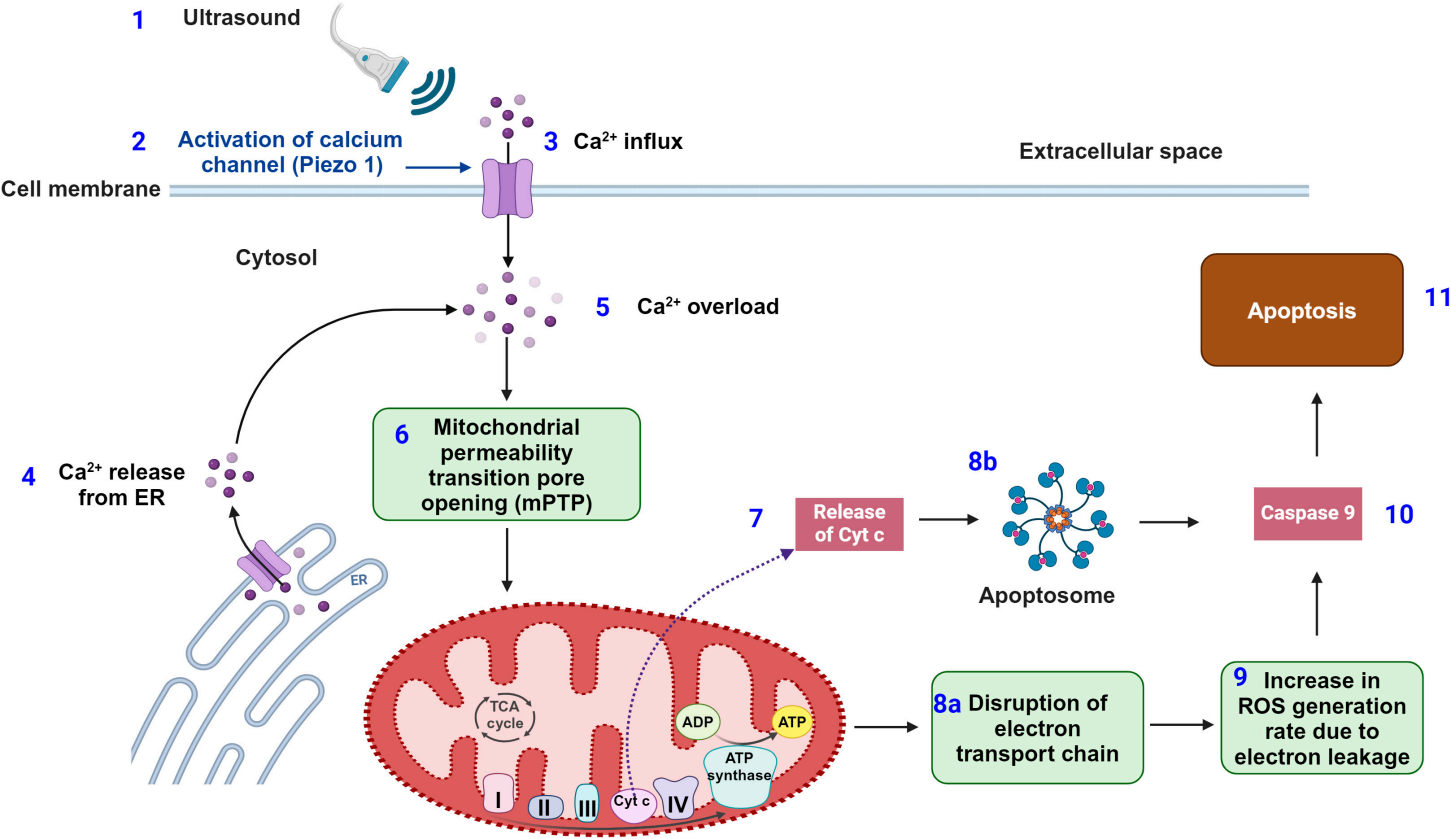
Mechanical pathway for ROS generation during SDT. (1) Application of ultrasound. (2) Mechanical force activates the cation channel (Piezo 1). (3) Ca^+2^ ions enter into the cytosol from extracellular space. (4) Endoplasmic reticulum (ER) also releases Ca^+2^ ions into the cytosol. (5) Increase in Ca^+2^ concentration leads to calcium overload. (6) Calcium overload opens the mPTP in the IMM. (7) This leads to the release of cytochrome c (Cyt c). (8a) The process results in disruption of the electron transport chain or (8b) activates enzymes such as apoptosomes. (9) Disruption of the electron transport chain increases electron leakage, and the ROS concentration consequently increases. (10 and 11) Caspase 9 is released, which induces programmed cell death or apoptosis.

This higher ROS concentration can damage mitochondrial DNA (mtDNA). The mtDNA damage, in turn, damages mtRNA transcription proteins responsible for the electron transport chain and, consequently, disrupts the electron transport chain. This process leads to a further increase in ROS generation and results in lower adenosine triphosphate (ATP) production and depolarization of the mitochondrial membrane potential (∆ψ_*m*_ ∼ −160 mV) [53]. These developments finally trigger the cell death, or apoptosis, pathway. A schematic diagram of mechanosensitive calcium channel opening due to ultrasound followed by the onset of the apoptotic pathway for tumor cell death is presented in Fig. 6.

#### Ultrasound-mediated microbubble dynamics

Depending on the amplitude of the applied ultrasound pressure during SDT, endogenous or exogenously administrated microbubbles can undergo either stable oscillation (SO) or inertial cavitation (IC). A relatively low mechanical index ($P_{a}/\sqrt{f}$), typically <0.4 [49], attributed to the low ultrasound pressure amplitude, leads to SO of the microbubble. In the case of SO, the bubble oscillates between its maximum and minimum radius around a mean radius. As a consequence of the volume change of the microbubble during SO, the surrounding liquid flow causes microstreaming [54]. The microbubble presence near the plasma membrane, therefore, can impose shear stress (100 to 1,000 Pa) [54] on the plasma membrane due to the microstreaming depending on the ultrasound pressure amplitude.

This shear stress generation due to liquid microstreaming can lead to the formation of transient pores [54–57] at the cell membranes. The membrane pores, of the order of a few nanometers to hundreds of nanometers formed during SO, are transient and become resealed over milliseconds to seconds [54,58] once the ultrasound application ceases. At a higher mechanical index (>0.4), however, the larger change in bubble volume causes rapid acceleration of the surrounding liquid toward the bubble in the form of a liquid microjet, resulting in violent bubble implosion and fragmentation. This is often termed as IC of the microbubble.

The potential energy released during bubble implosion can damage the cell membrane and rupture the cytoskeleton, either in the proximity of the microbubble or from a distance [55]. The force exerted by the liquid microjet during IC is higher than during an SO event, which might even result in the permeabilization of the blood vessels. The pore size formed during IC is reported to be higher at hundreds of nanometers to the micrometer range [54] than for pores created during an SO event. The resealing time for membrane pores generated during IC is larger at tens of seconds and may be irreparable [55,59].

**Fig. 7.**
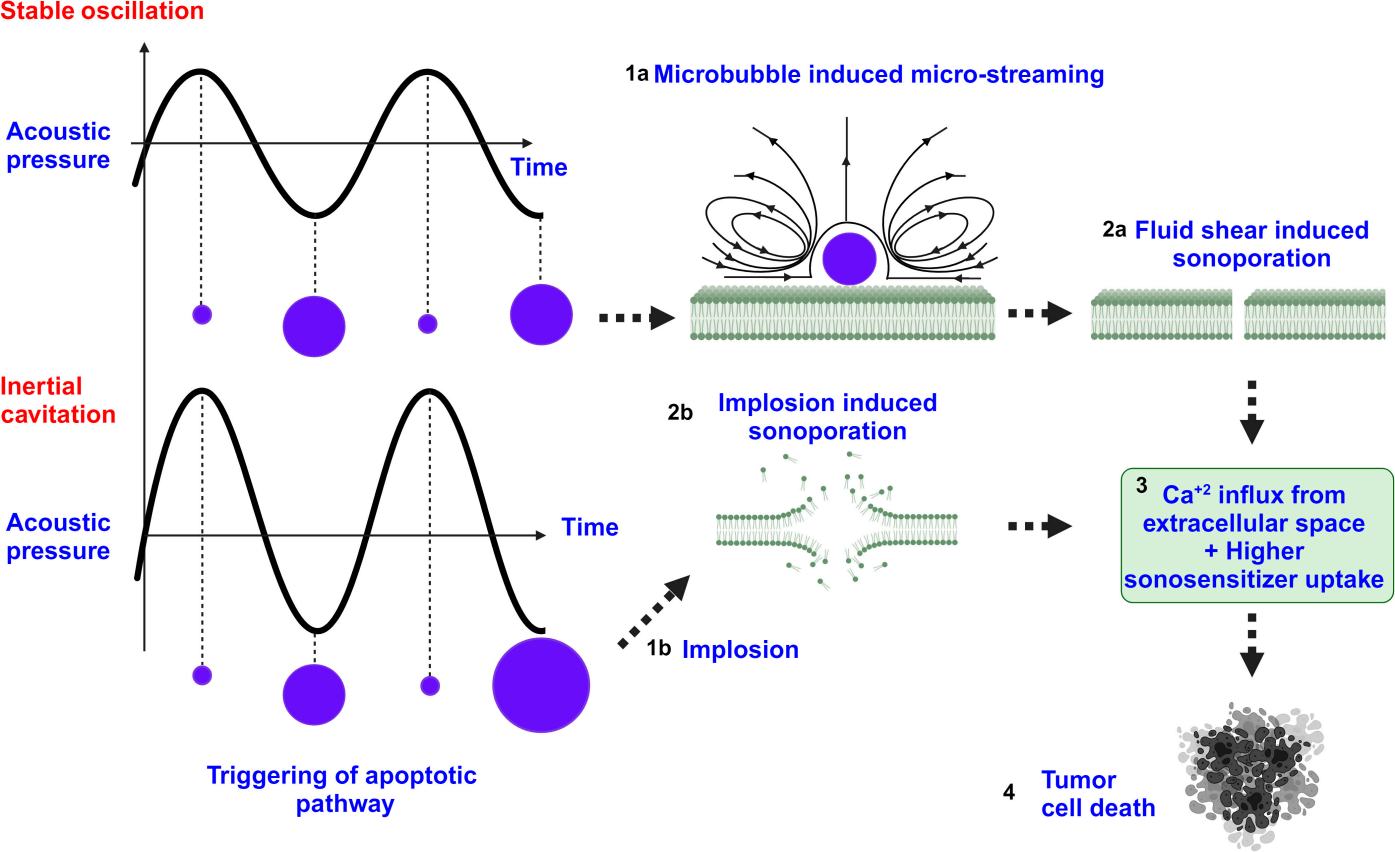
Schematic of sonomechanical pathway in the presence of a microbubble during SDT. At a lower ultrasound pressure, the microbubble undergoes SO, whereas at a higher ultrasound pressure, IC occurs. (1a) SO mediated microstreaming. (1b) Bubble implosion. (2a) Microstreaming-induced fluid shear leads to the formation of transient pores at the plasma membrane. (2b) Implosion-induced sonoporation at the plasma membrane. (3) Sonoporation allows both Ca^+2^ influx from the extracellular space into the cytosol and higher sonosensitizer uptake by the tumor cells. (4) Triggering of cytotoxic pathways via Ca^+2^ overloading and sonosensitizer activation.

Pore formation in the plasma membrane of tumor cells during either an SO or IC event can allow Ca^+2^ influx into the cytosol as well as endocytosis of large molecules, such as sonosensitizers and chemotherapeutic drug molecules [54,60]. The higher Ca^+2^ influx can disrupt calcium homeostasis between cytosol and the mitochondria, which can disrupt the electron transport chain, followed by an increase in ROS generation and oxidative stress. Higher cellular uptake of the sonosensitizer through membrane pores can also increase antitumor activity, enhancing SDT efficacy [60]. A schematic diagram of microbubble-assisted sonomechanical pathway activation during SDT is shown in Fig. 7.

While it may also be possible that, depending on their activation energy, sensitizers absorb ultrasound energy directly without cavitation to become excited, there is no experimental evidence in the literature. To enhance SDT efficacy, further investigation of sensitizer activation, ultrasound–sensitizer interaction, and energy transfer is required.

## Ultrasound Details for SDT

### Selection of transducer: Focused versus unfocused transducer

Choosing between focused and unfocused transducers is critical for SDT efficacy. Unfocused transducers distribute the ultrasound energy more broadly, which can be advantageous for treatments requiring coverage over a larger area but may result in lower intensity and reduced precision. Focused transducers concentrate ultrasound energy in a specific focal zone, producing higher intensity at that zone but minimizing exposure to its surroundings. Since this energy concentration enhances the therapeutic effect and improves precision while targeting lesions, it is often the preferred method for SDT.

The following relations describe the beam width (BW) and depth of focus (DOF) at −6 dB:

$$\mathrm{BW}=1.44\times F^{2}\times \lambda$$ (1)

$$\mathrm{DOF}=9.68\times F^{2}\times \lambda$$ (2)

where *F* denotes the ratio of the focal length of the transducer to the diameter of the active element and *λ* is the wavelength.

### Ultrasound parameters

#### Frequency

Ultrasound frequency [61] measured in hertz refers to the number of mechanical oscillations occurring per unit time where, for instance, a 3-MHz frequency implies 3 million cycles per second. For SDT applications, ultrasound frequencies typically range from 0.5 to 3 MHz, a lower range than those used during diagnostic ultrasound, which lie from 5 to 20 MHz. Lower SDT frequencies allow more time for cavitation bubbles to grow, resulting in substantial energy release when they collapse. More importantly, using relatively lower frequencies during SDT enhances its superiority over PDT, which has extremely limited penetration.

There is an inverse relationship between ultrasound frequency and attenuation or penetration through a medium. Higher frequencies are associated with higher attenuation and lower penetration depth. For example, 1-, 3-, and 5-MHz ultrasound frequencies can penetrate up to 4, 3, and 0.5 cm, respectively.

Attenuation, representing energy loss, also varies with the medium. For instance, bone has a high attenuation coefficient (5 dB/cm at 1 MHz), while water has a much lower attenuation coefficient (0.002 dB/cm at 1 MHz). This variability highlights the importance of selecting appropriate frequencies for effective SDT treatment, ensuring adequate penetration and energy delivery to target tissues.

#### Intensity

Intensity (*I*) [62] is a critical parameter in ultrasound applications, representing the power per unit area, and is typically expressed in W/cm^2^, i.e.,

$$I=\frac{P}{A}\;$$ (3)

where *P* denotes the power of the ultrasound beam in watts and *A* is the cross-sectional area in square centimeters.

There are several types of intensity measurements, each providing different information about the ultrasound beam. Spatial peak intensity (*I*_SP_) measures the maximum intensity at a specific point within the beam, while spatial average intensity (*I*_SA_) calculates the average intensity over the entire cross-sectional area of the beam. Temporal characteristics also play a role, with temporal peak intensity (*I*_TP_) representing the peak intensity during the pulse and temporal average intensity (*I*_TA_ providing the average intensity over the entire pulse cycle. Most therapeutic applications [63] operate ultrasound in pulse mode to minimize the heating effects on both the medium and the transducer’s active element.

The spatial peak pulse average intensity (*I*_SPPA_) becomes an essential parameter in this context. *I*_SPPA_ is defined as the ultrasound wave’s peak intensity averaged over a single pulse’s duration. It represents the maximum intensity within the beam during the active pulse period, measuring the peak energy delivered to the tissue in each pulse. *I*_SPPA_ is calculated as follows:

$$I_{\text{SPPA}}=\frac{P_{\text{peak}}^{2}}{2\rho c}$$ (4)

where *P*_peak_ is the peak acoustic pressure at the focal zone of the transducer, *ρ* is the material density, and *c* is the speed of sound in the medium.

However, for long-term exposure and overall energy delivery, the spatial peak temporal average intensity (*I*_SPTA_), which indicates the highest intensity average over time, is the more critical parameter for an SDT application. *I*_SPTA_ accounts for the duty cycle of the ultrasound wave, providing a measure of the average intensity over time, including both the active pulse duration and the silent intervals between pulses.

The relationship between these intensities is [64]

$$I_{\text{SPTA}}=I_{\text{SPPA}}\times \mathrm{DF}$$ (5)

where the duty cycle (*DF*) is the fraction of time the ultrasound is actively transmitting, defined as the ratio of pulse duration (*PD*) to the pulse repetition time (*PRT*),

$$\mathrm{DF}=\frac{\mathrm{PD}}{\mathrm{PRT}}$$ (6)

It can also be expressed in terms of the pulse repetition frequency (*PRF*), which is the number of pulses per second:

DF=PD×PRF (7)

For example, if *I*_SPPA_ is 1 W/cm^2^ (measured by hydrophone), and a 10% duty cycle was set during the experiment, then *I*_SPTA_ = 1 × 0.1 = 0.1 W/cm^²^.

**Fig. 8.**
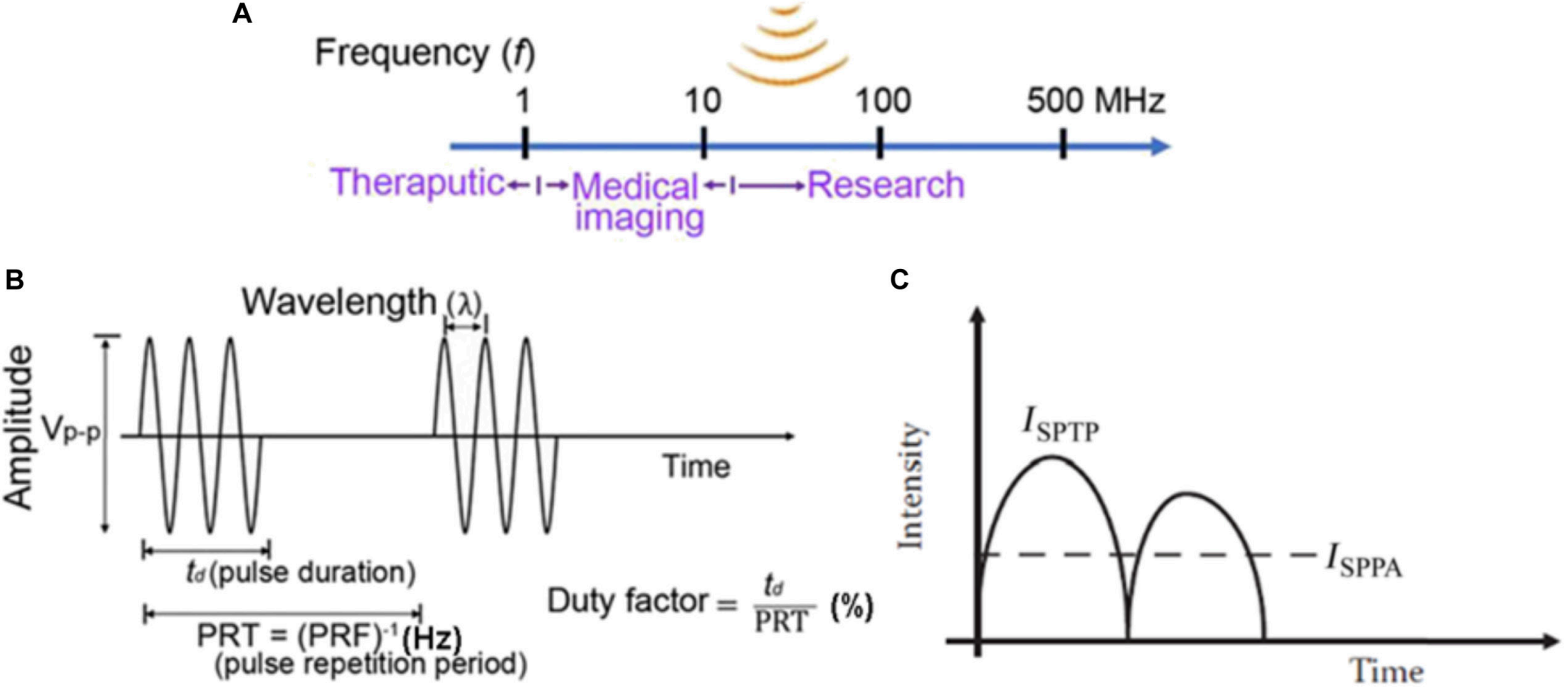
(A) Schematic representation of sound waves at different frequencies and corresponding application areas. (B) Critical ultrasound parameters, which are commonly used in SDT and for other application areas. This image is reproduced with permission from [65], Copyright 2021, SAGE Publications. (C) Schematic diagram of *I_SPPA_*, adapted from [62].

The intensity of SDT application must be carefully controlled to balance efficacy and safety. Higher intensities can have more impactful therapeutic effects, including killing cancerous cells, but they also increase the risk of thermal and mechanical damage to surrounding tissues. For this reason, most SDT applications employ intensities ranging from 0.5 to 3 W/cm^2^, ensuring sufficient energy delivery without excessive heating. Figure 8 is a schematic representation of fundamental ultrasound parameters.

The interplay between frequency and intensity is critical in designing effective SDT protocols. Lower frequencies combined with appropriate intensities can achieve deeper penetration and effective energy delivery, making them ideal for treating less accessible lesions. The choice of frequency and intensity must consider the specific properties of the tissue, such as its attenuation coefficient and density, to ensure precise targeting and optimal therapeutic outcomes. The selection of frequency and intensity in SDT is a tailored process that depends on the therapeutic goals, tissue characteristics, and the need to balance efficacy with safety. By understanding and optimizing these parameters, clinicians can enhance the effectiveness of SDT, providing a promising approach for treating various types of cancer and other diseases while minimizing potential risks.

#### Coupling material

A key concern for SDT is the impedance mismatch between the ultrasound transducer and the targeted subject. Acoustic impedance, defined as the product of a material’s density (*ρ*) and the speed of sound (*c*) in the material, varies substantially for different materials. A large difference in impedance between the transducer, typically made of a piezoelectric crystal material, and the target leads to a substantial portion of the ultrasound energy being reflected at the interface rather than transmitted into the target, leading to unsatisfactory therapeutic outcomes.

To address this issue, coupling gels are applied to bridge the gap between the transducer and the treated sample, enhancing the transmission of ultrasound waves. The most commonly used coupling gels in SDT and ultrasound applications are typically water-based due to their similarity in acoustic impedance to human tissue (approximately 1,500 m/s), which minimizes reflection and maximizes energy transfer.

## Recent Advances in SDT for Cancer Treatment

### SDT for glioblastoma/grade IV astrocytoma

Most preclinical SDT studies have treated glioblastoma multiforme (GBM) or grade IV astrocytoma, which are lethal, deep-tissue brain tumors with a very high mortality rate [66]. While SDT is primarily dependent on ROS generation, which triggers cellular death pathways, its efficacy can be deterred by tumor hypoxia. High glutathione (GSH) concentration in the TME consumes singlet oxygen (^1^O_2_), lowering SDT efficacy. The BBB also limits SDT-based GBM treatment [14].

**Fig. 9.**
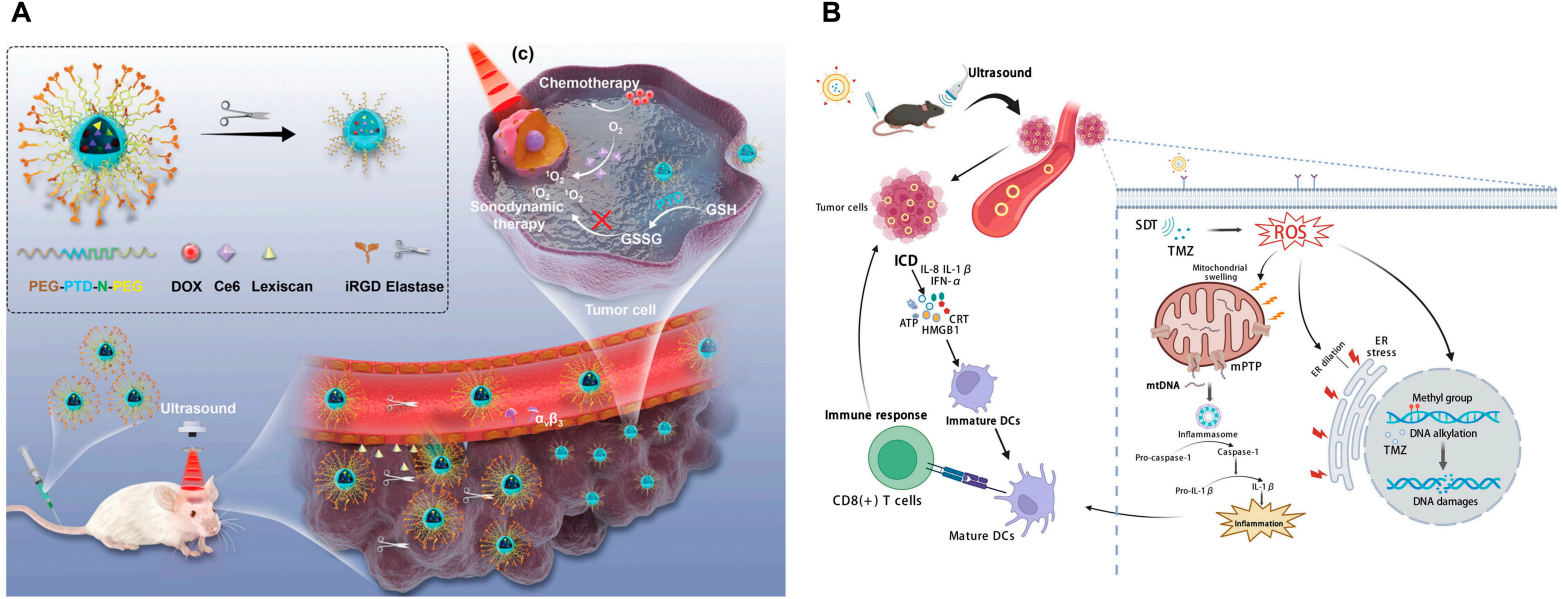
Schematic representation of the current SDT-based multimodal therapeutic approach for GBM. (A) Chlorin e6-assisted SDT combined with Lexiscan-loaded poly (2,2″-thiodiethylene 3,3″-dithiodipropionate) nanoparticles for doxorubicin delivery in brain tumors in vivo. The SDT-chemotherapy combined therapy shows an improvement in the efficacy via depletion of GSH. This image is reproduced with permission from [67]. https://creativecommons.org/licenses/by/4.0/ (B) TMZ is used as a sonosensitizer, which causes mitochondrial membrane permeabilization and ER stress. This induces mtDNA release within the cytoplasm and triggers the immunogenic signal [e.g., interleukin-1β (IL-1β)] that facilitates dendritic cell activation followed by glioma cell death. This image is reproduced with permission from [72], Copyright 2023 Elsevier Inc.

To address these challenges, SDT-based multimodal therapy combines SDT with temozolomide (TMZ)/bevacizumab/doxorubicin-based chemotherapy [67,68], with PDT [69], and with immunotherapy [14,15,67]. Recent focus has also been placed on using the existing Food and Drug Administration (FDA)-approved chemotherapy drug TMZ as a sonosensitizer [70,71] since TMZ damages DNA bases through methylation and can cause ROS-mediated mitochondrial damage due to its sensitizing capability, enhancing SDT therapeutic efficacy. A summary of recent progress on SDT-based multimodal therapeutic approaches for GBM is presented in Fig. 9.

### SDT for breast cancer

**Fig. 10.**
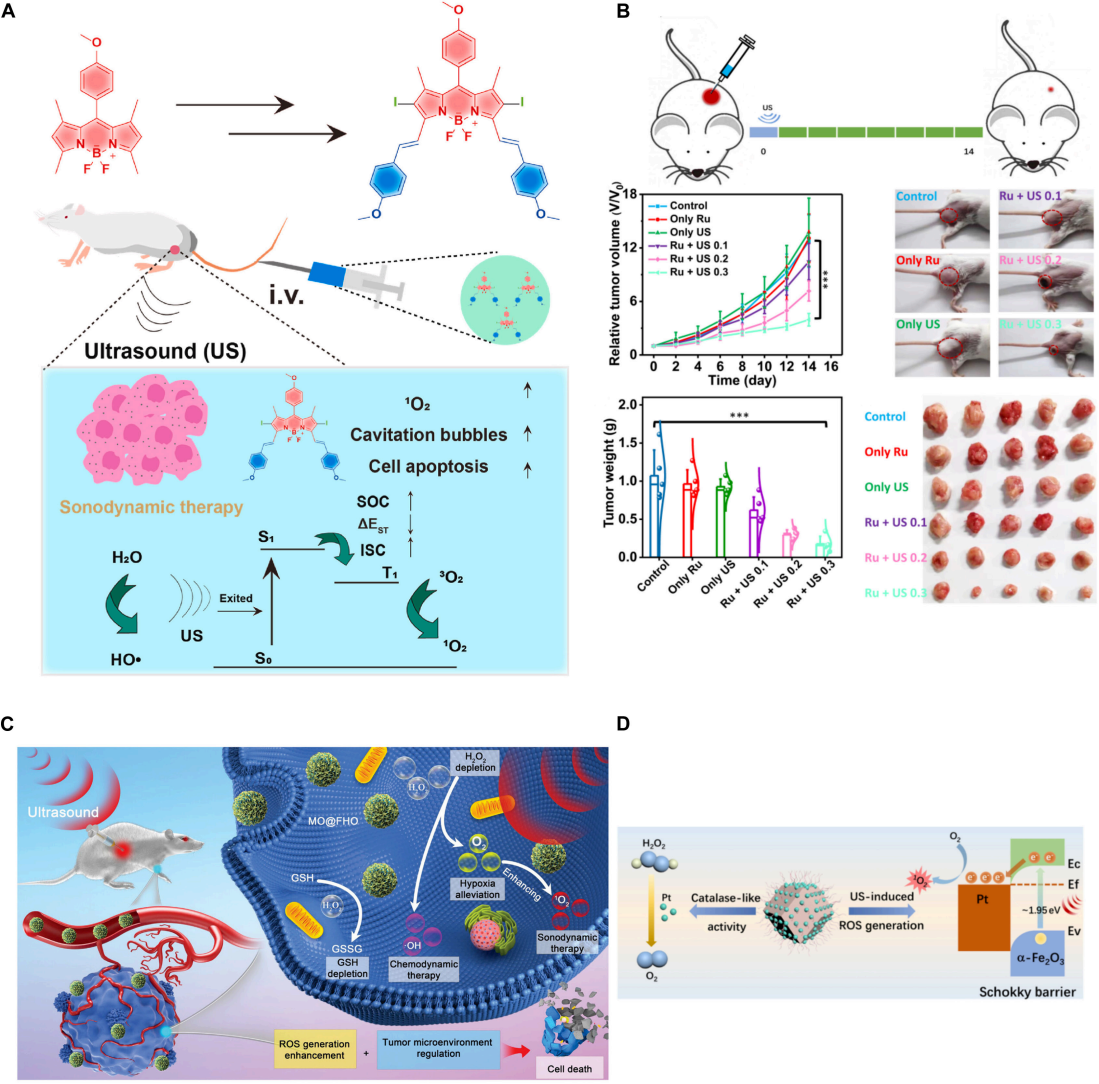
Recent advances of sonosensitizers in SDT-mediated BC therapy. (A) For the first time, 4 BODIPY derivative sonosensitizers are used for treating breast tumors (4T1 cell line). The sensitizer with the lowest singlet to triplet state transition energy gap (1.1243 eV) exhibits the highest antitumor effect in vivo. This image is reproduced with permission from [74], Copyright 2023 Elsevier Masson SAS. (B) [Ru(bpy)_3_]^2+^ is used as a novel sonosensitizer for treating 4T1 tumor-bearing mice. [Ru(bpy)_3_]^2+^ has a very low energy gap (0.1239 eV) between LUMO and HOMO, which leads to its activation by ultrasound stimulation (0.1 to 0.3 W/cm^2^, 3 MHz, 20-min stimulation) and, thereby, generates cytotoxic singlet oxygen. Further, during SDT, NADPH to NAD^+^ oxidation causes redox imbalance in the TME, which results in an arrest of tumor growth. This image is reproduced with permission from [75]. https://creativecommons.org/licenses/by/4.0/ (C) FeOOH-MnO_2_ nanocomposite exhibits dual features. It inhibits the electron-hole pair recombination and, hence, increases ROS generation (singlet oxygen and hydroxyl radicals). Additionally, due to its catalytic activity, H_2_O_2_ to O_2_ decomposition causes hypoxia alleviation in the TME and GSH depletion. This collectively enhances ROS generation rate and SDT efficacy in mice bearing BC. This image is reproduced from [77]. https://creativecommons.org/licenses/by/4.0/ (D) α-Fe_2_O_3_ with Pt nanocrystal-based photosensitizer, which reduces the energy gap and enhances ROS generation, followed by an increase in SDT efficacy in 4T1 tumor-bearing mice. This image is reproduced from [78]. https://creativecommons.org/licenses/by/4.0/

SDT-based breast cancer (BC) investigations, still in the preclinical stage, use both organic and inorganic sonosensitizers with a multimodal approach to enhance therapeutic efficacy [22,73]. Novel biodegradable sonosensitizers that have higher ROS generation efficiency, high molar absorption, and lower energy interval between LUMO (lowest unoccupied molecular orbital) and HOMO (highest occupied molecular orbital) and exhibit low cytotoxicity in healthy cells have been developed. Here, boron-dipyrromethene (BODIPY) [74], ruthenium-based metal complexes [75], a monosulfide nanoparticle-based sensitizer [76], composite sonosensitizers (FeOOH-MnO_2_ [77], α-Fe_2_O3 with Pt nanocrystals [78]), and gas-assisted sensitizers [79] have shown promising outcomes for SDT-mediated BC treatment. Figure 10 presents a schematic of recent strategies for SDT-based BC treatment.

### SDT for pancreatic cancer

**Fig. 11.**
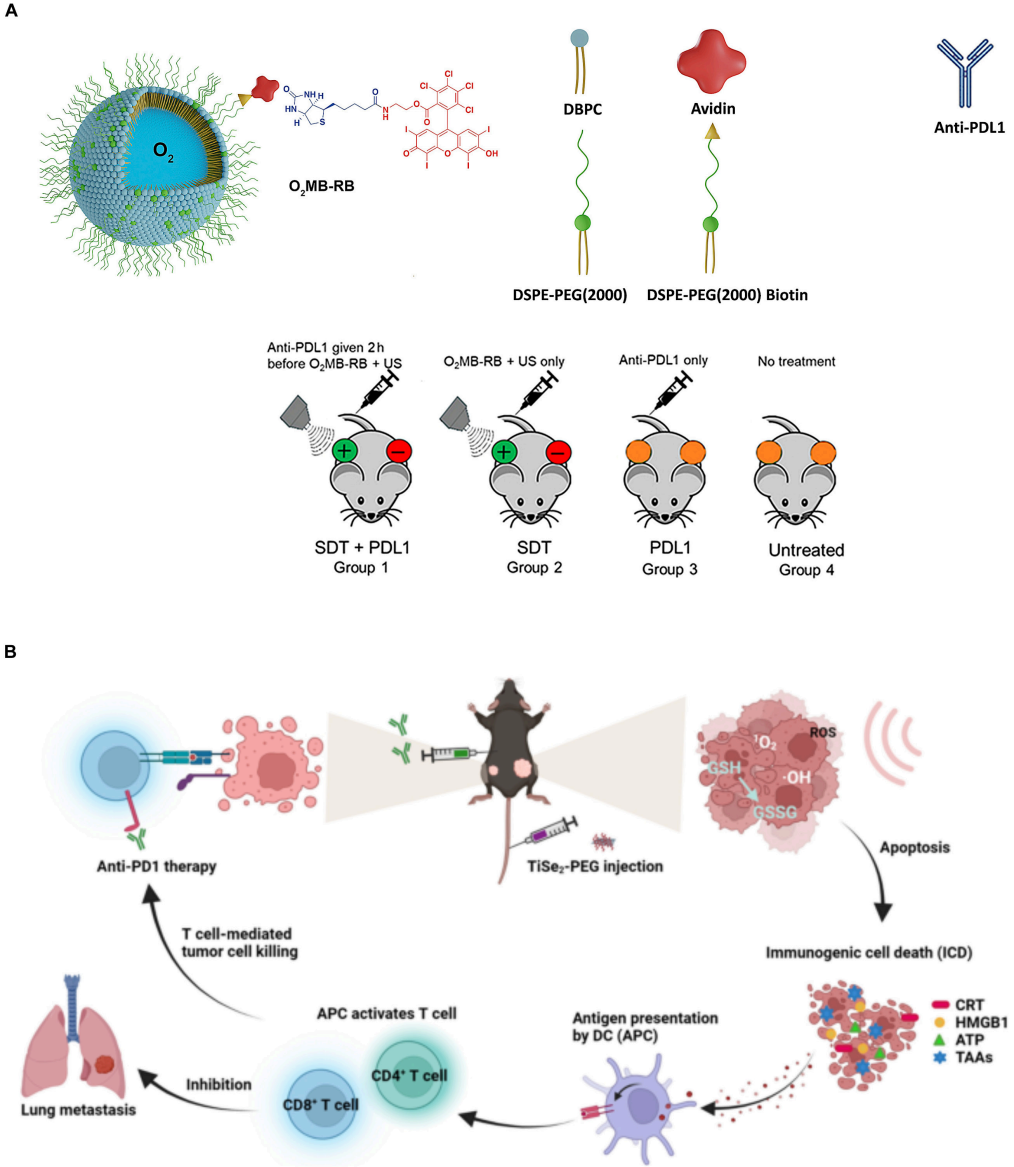
SDT-based multimodal therapy for treating pancreatic cancer. (A) O_2_-microbubble loaded with Rose Bengal sensitizer is used in combination with anti-PDL1. The combined therapy showed the maximum antitumor effect on pancreatic cancer tumor-bearing mouse models compared to SDT as well as immune checkpoint inhibitor alone. This image is reproduced with permission from [24], Copyright 2021 Elsevier B.V. (B) SDT is combined with anti-PD1 with TeS_2_ nanosheets as a sensitizer. SDT induces an immunogenic signal that enhances tumor-associated antigen and cytokine release, followed by dendritic cell activation. This ultimately enhances CD8^+^ infiltration and therapeutic efficacy. This image is reproduced with permission from [82]. https://creativecommons.org/licenses/by/4.0/

Pancreatic cancer is a deep-tissue solid tumor with a very low 5-year survival rate after diagnosis of ~10% [80]. SDT-based pancreatic cancer treatment is still in the preclinical stage as the heterogeneous TME, dense stroma, hypoxic core, and high GSH concentration (~10 mM) lower SDT efficacy [80]. Recent attempts to overcome these challenges include the design of novel sensitizers, administering O_2_ microbubbles, and combining SDT with immunotherapy. The recent SDT strategies for pancreatic cancer treatment are shown in Fig. 11.

To alleviate tumor core hypoxia, hollow mesoporous organosilica nanoparticles loaded with the IR780 sensitizer have been used as a self-O_2_ production nanoplatform [81]. Titanium diselenide (TiSe_2_) nanosheets are used as a sonosensitizer and combined with anti-PD1 immune checkpoint blockade for treating pancreatic cancer in hypoxic and normoxic conditions [82]. TiSe_2_ produces a large amount of ROS upon ultrasound stimulation and induces immunogenic cell death, followed by a release of tumor-associated antigens, which activate dendritic cells and increase CD8^+^ infiltration.

In addition to the use of inorganic sensitizers, conventional organic sensitizer-based multimodal therapy has also been employed [23]. SDT has used Rose Bengal conjugated with O_2_ microbubbles and the anti-PDL1 ligand to treat pancreatic cancer. This SDT-based multimodal approach showed maximum antitumor effect by enhancing CD8^+^ and CD4^+^ infiltration over SDT immunotherapy alone [24].

### SDT for hepatocellular carcinoma

**Fig. 12.**
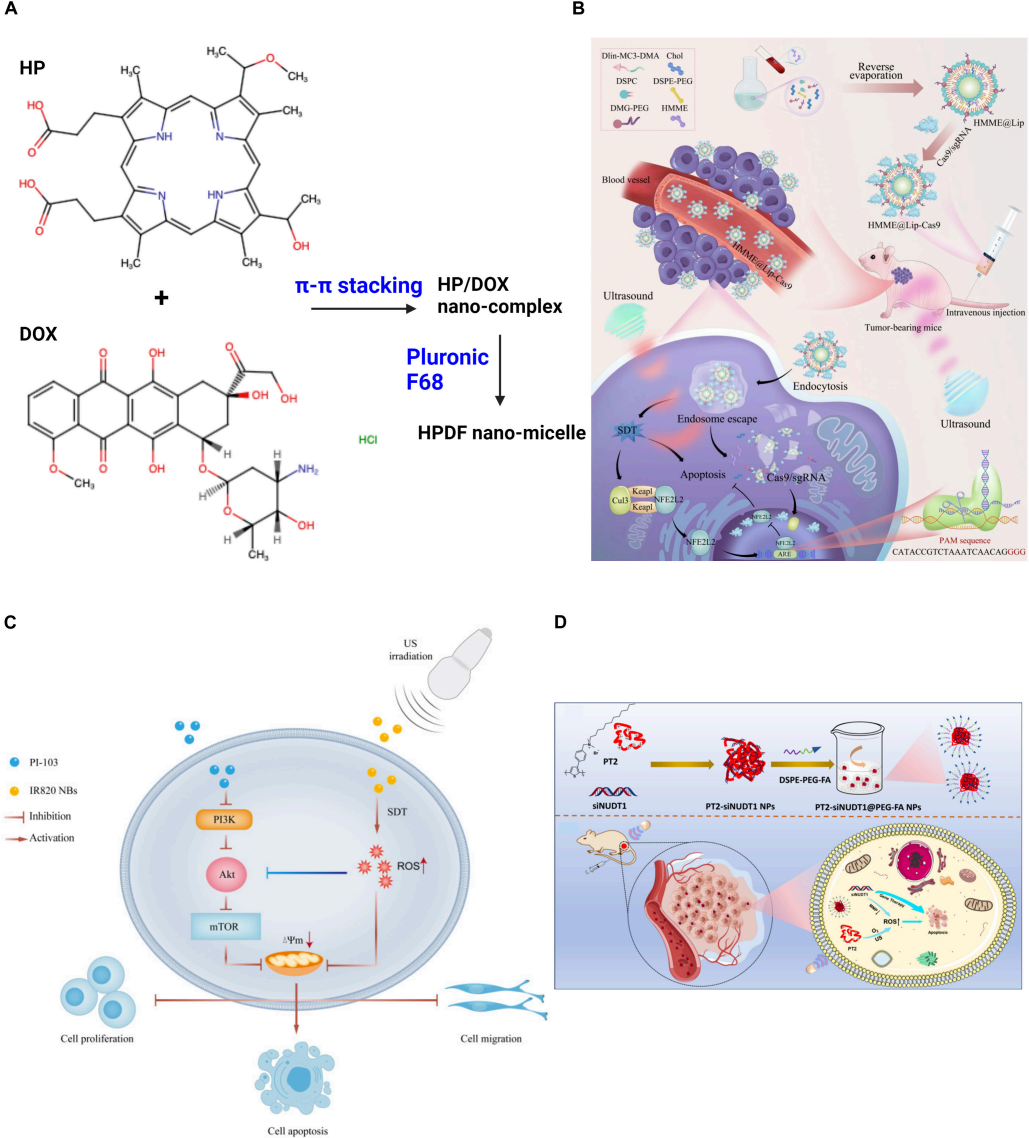
Multimodal SDT for HCC treatment. (A) HMME sensitizer is combined with doxorubicin for SDT-chemotherapy. Redrawn from [86] using Biorender. (B) Combination of Cas9/single-guide RNA (sgRNA) with HMME shows a promising antitumor effect due to the knockdown of the NFE2L2 transcription factor, which plays an important role in the reduction of oxidative stress and prevents cell damage. This image is reproduced with permission from [87]. https://creativecommons.org/licenses/by-nc-nd/4.0/ (C) The application of IR820 sensitizer dye within the NB in combination with PI-103 shows a synergistic effect due to its inhibitory nature in the PI3K/ mTOR signaling pathway. This image is reproduced with permission from [88]. http://creativecommons.org/licenses/by-nc/3.0/. (D) si-NUDT1 is used with polythiophene, which shows a substantial antitumor effect due to the silencing of the NUDT1 gene and the singlet oxygen as well as ROS generation during ultrasound application. This image is reproduced (adapted) with permission from [89], Copyright 2024, American Chemical Society.

Hepatocellular carcinoma (HCC), or liver cancer, ranks third in cancer-related death [83]. HCC is a deep-tissue tumor with a poor prognosis, leading to challenges in surgical resection, and often exhibits chemoresistance via Bcl-2- and Bcl-XL-mediated resistance against cellular apoptosis [84,85]. SDT could, therefore, be an alternative therapeutic approach for treating HCC (Fig. 12) and is currently in the preclinical stage.

Recent SDT investigations for HCC have been primarily focused on a multimodal approach to increase the therapeutic efficacy. Nanobubble (NB)-assisted SDT has shown promise, where NBs conjugated with the FDA-approved ultrasound dye indocyanine green showed a substantial antitumor effect through the necroptosis pathway [84]. The near-infrared dye IR820 is also used as a sonosensitizer in conjunction with perfluoropropane-filled NBs due to the inhibitory nature of PI-103 in the phosphatidylinositol 3-kinase (PI3K) and mammalian target of rapamycin (mTOR) signaling pathway for HCC [88]. Ultrasound-responsive multifunctional nanoparticles have also gained attention for an SDT-chemotherapy-based HCC treatment [86,90].

The combination of SDT and gene therapy is another current focus, where targeting and knocking down specific transcriptional factors such as NFE2L2 (nuclear factor erythroid 2-related factor 2) using CRISPR/Cas9 is employed [87]. The ultrasound stimulation in the presence of the hematoporphyrin monomethyl ether (HMME) sensitizer produces singlet oxygen. This leads to cell membrane lipid peroxidation and, consequently, the release of Cas9 within the nucleus, which finally knocks down NFE2L2 and improves SDT efficacy. Small interfering RNA (siRNA), such as si-NUDT1 in combination with polythiophene, has also been used for HCC treatment. A synergistic effect is observed, where polythiophene generates singlet oxygen and ROS under ultrasound stimulation, and the use of si-NUDT1 silences the antioxidative stress effect caused by the NUDT1 gene, leading to a promising antitumor effect [89].

### SDT for prostate cancer

**Fig. 13.**
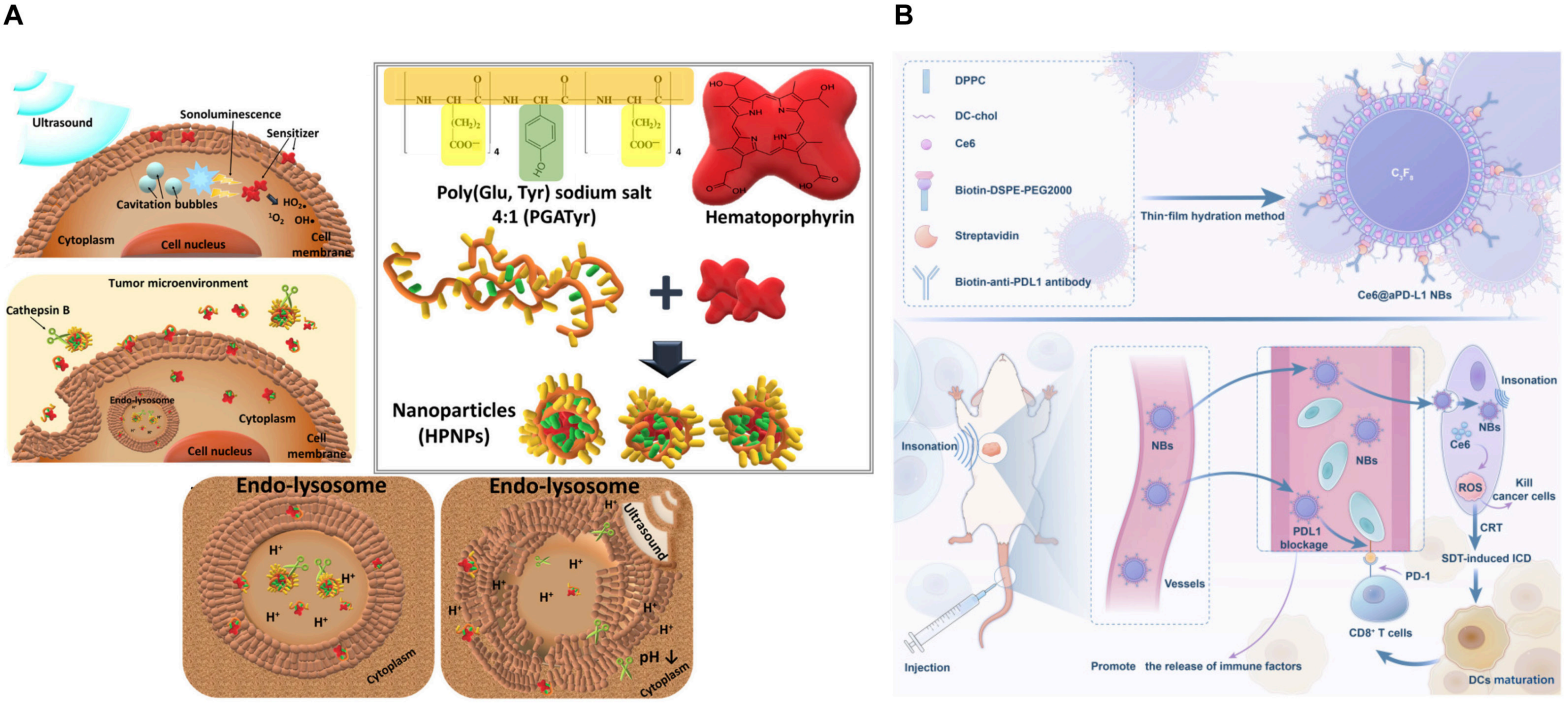
SDT for PC treatment. (A) Hematoporphyrin conjugated with glutamate and tyrosine via hydrophobic and *π* − *π* interactions, forming a self-assembled nanoparticle HPNP. The acidic TME, along with the presence of cathepsin B, causes the digestion of the HPNP, which reduces its size. This allows higher accumulation of the sensitizer molecule due to an increase in its diffusion within the tumor cell and promising therapeutic efficacy in terms of reduction of the tumor volume within 24 h after the dose administration in vivo. This figure is reproduced from [27]. https://creativecommons.org/licenses/by/4.0/. (B) SDT-based multimodal therapeutic approach has been adopted. Ce6, along with anti-PDL1, is encapsulated within the NB. The ultrasound stimulation enhances ROS generation, and the presence of an immune checkpoint inhibitor, anti-PDL1, enhances immune response, which finally leads to immunogenic cell death. This figure is reproduced with permission from [91]. http://creativecommons.org/licenses/by-nc/3.0/

Prostate cancer (PC) treatment using SDT has been sparse compared to other deep-tissue solid tumors and is in the preclinical stage (Fig. 13). The hematoporphyrin sensitizer molecule is conjugated with glutamate and tyrosine to form ultrasound-responsive nanoparticle HPNP (hematoporphyrin-containing PGATyr-based nanoparticles). The presence of cathepsin B, of acidic nature in the TME, induces digestion and consequently a size as well as charge reduction of the HPNP. This promotes higher diffusion of the sensitizer molecule, thereby increasing its accumulation within the tumor cells. A substantial reduction in the tumor volume (64%) is observed in the presence of ultrasound alone after 24 h of the dose being administered in vivo [27]. The SDT-based multimodal therapeutic approach has also gained traction for treating PC. Chlorin e6 and anti-PDL1 are encapsulated within lipid-coated NBs [91]. This multimodal approach shows an improvement in the immune response in vivo due to dendritic cell activation and CD8^+^ infiltration enhancement, which ultimately leads to immunogenic cell death.

## Sonosensitizers for SDT-Based Cancer Treatment in Preclinical and Clinical Trials

### Sonosensitizers in preclinical studies

The sonosensitizers used in preclinical SDT studies are both organic and inorganic molecules. The most studied organic sonosensitizers for GBM, triple-negative BC, PC, HCC, and pancreatic cancer are porphyrin based [e.g., 5-aminolevulinic acid (5-ALA) and hematoporphyrin]. Xanthene-based sensitizers (e.g., Rose Bengal), anti-inflammatory drugs (e.g., methylene blue), and, more recently, the FDA-approved antitumor drug TMZ for GBM are also used as organic sonosensitizer molecules for various cancer treatments using SDT.

These organic sonosensitizers can also be activated with light. Thus, they may exhibit phototoxicity and skin sensitivity. Organic sensitizers also suffer from poor chemical stability [92]. Here, inorganic sensitizers have improved chemical stability and, consequently, have better therapeutic efficacy [5]. Therefore, efforts have been made to develop nanoparticle-based sensitizers, supramolecular sensitizers, and metal–organic framework (MOF)-based sensitizers.

The details of different classes of organic and inorganic sonosensitizers, along with the ultrasound parameters (frequency, intensity, duty factor, pulse repetition frequency, and stimulation duration) for SDT preclinical studies (in vivo and in vitro) of various cancers are listed in Table S1 (see supplementary file).

### Sonosensitizers in clinical trials

The state-of-the-art current clinical trials for SDT are focused on brain tumor treatments, such as recurrent glioblastoma, high-grade glioma, and diffuse intrinsic pontine glioma [11,93]. 5-ALA is a clinically approved drug molecule for use in glioma surgery [94] due to its ability to cross the BBB, selective accumulation within the tumor cells, and fluoresce upon activation via light. Preclinical animal glioma studies reveal that 5-ALA is converted to protoporphyrin IX (PpIX), which is activated by the energy released from sonoluminescence, generating ROS, with results in tumor cell death [16].

In neuro-oncology clinical trials, 5-ALA is used in combination with low-intensity focused ultrasound (FUS). The first human phase 0 clinical trial (NCT04559685) reported that SDT with 5-ALA HCl (SONALA-001) is safe for use in high-grade glioma and has no off-target cellular effects. In this study, ultrasound was applied to half of the tumor volume with varying energy (200 to 800 J) with a magnetic resonance-guided FUS (220 kHz) after 5 to 7 hours of SONALA-001 administration intravenously. The outcome revealed that the SDT-treated tumor volume exhibited higher oxidative stress compared to the untreated tumor volume and, hence, confirms the direct evidence of ROS generation during SDT. A dose escalation and expansion of this study is ongoing (NCT05370508, phase 1/2) [11].

A phase 2 clinical trial (NCT04845919) used a similar ExaAblate model 4000 type-2 neuro system with oral administration of 5-ALA in SDT. A similar magnetic resonance-guided FUS device (MRgFUS) is being used in a phase 1/2 clinical trial (NCT05123534) for treating diffuse intrinsic pontine glioma in children with SONALA-001, where the main focus is determination of the safety and dose tolerability of the treatment.

The ongoing phase 1 clinical trial NCT06039709 uses a neuronavigation-guided LIPU to stimulate 50% of the recurrent GBM tumor volume (6 to 20 cm^3^) 6 hours after 5-ALA administration orally. The primary focus of this study is also the safety and feasibility of SDT. Clinical trial NCT05362409 uses a different system, CV-01, for delivering the ultrasound energy diffusely over the brain to treat high-grade glioma with ALA. The ultrasound stimulation duration and assessment of its tolerability are the primary foci of this study [6,11,95].

Hematoporphyrin is a porphyrin-based sensitizer reported in a recent SDT clinical trial (ChiCTR2200065992) for recurrent glioblastoma treatment [19]. Despite showing potential, the long-term survival benefit of this study cannot be concluded due to the limited number of participating patients.

**Fig. 14.**
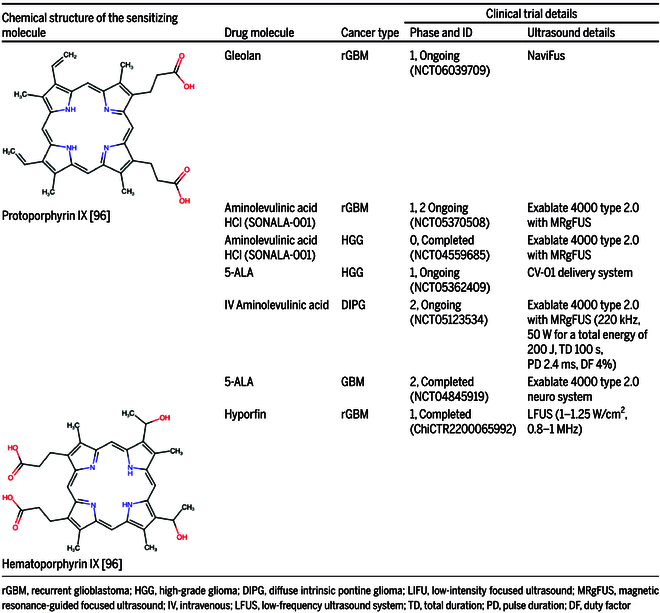
Porphyrin sonosensitizers for SDT-based clinical trials [96].

A summary of SDT clinical trials based on the porphyrin sonosensitizers is provided in Fig. 14.

### Future outlook for clinical trials

Preclinical SDT studies on different types of cancer and their translation to human clinical trials for brain tumors reveal that SDT is safe and has the potential to treat deep-tissue solid tumors. However, the therapeutic efficacy of SDT depends on the ultrasound parameters, especially frequency, intensity, and stimulation duration, since these parameters drive sonosensitizer activation that triggers tumor cell death. Therefore, extensive additional preclinical experiments are required to optimize tumor-specific ultrasound parameters. It has been established that ROS-mediated enhanced oxidative stress is the primary cause of tumor cell death through SDT, but the exact path (such as necroptosis and ferroptosis along with the known apoptotic pathway) for tumor cell death during SDT is still unknown. This lack of knowledge must be addressed to improve SDT efficacy for various types of solid cancer treatments.

## Outlook and Future Directions

In this review, we discussed recent developments in SDT-based deep-tissue cancer treatments. It is evident that SDT is still in the preclinical stage for most solid tumor treatments, and there is ample scope for improving its therapeutic efficacy and developing successful cancer therapies. Therefore, in the following sections, we discuss the key strategies required to overcome current challenges facing SDT. Addressing these challenges should pave the pathway to clinical translation for different types of solid tumor treatments.

### Roadmap for the clinical translation for SDT

#### New sonosensitizer development: HOMO-LUMO energy gap, spin-orbit coupling

Sonosensitizers are the central component of SDT. Therefore, designing an effective sensitizer molecule is the most important aspect of enhancing SDT efficacy. The often-used organic sensitizers, e.g., porphyrin sensitizers, have low water solubility, complex synthesis routes, lower chemical stability, lower sensitivity, and, thereby, low ROS yield. On the other hand, inorganic sensitizers have limitations in terms of their larger size, reduced accumulation in cancer cells, poor biocompatibility, and biodegradability. Therefore, new sensitizers with high energy absorption efficiency, lifetime, chemical stability, selectivity, bio-compatibility, and bio-degradability are the primary need for improving SDT efficiency.

The most acceptable hypothesis for sonosensitizer activation during SDT is the absorption of photons from sonoluminescence produced through ultrasound stimulation. Following photon absorption, the process involved in producing singlet oxygen from the perspective of a sensitizer can be described as follows.

1. Absorption of a photon from ground singlet (S_0_) produces an excited singlet (S_1_) state.
2. Excited singlet state undergoes ISC to excited triplet state that is generally long-lived.
3. The excited triplet state transfers the energy to oxygen, which is in its ground triplet state and produces cytotoxic singlet oxygen.

Sensitizers with high molar absorption coefficients ensure efficient absorption of photons, resulting in an electronic transition. Sensitizers that absorb at lower energies of the visible spectrum are desired for SDT. In sensitizers, the energy gap between the LUMO and the HOMO controls the absorption energy with different molecular architectures [97]. However, the rate of nonradiative energy transfer (*k_nr_*) increases exponentially with the decrease in the energy gap according to the energy gap law [98,99]. The increase in *k_nr_* leads to energy loss via kinetic energy transfer, or heat, which results in a decrease in the electron population in the singlet excited state and ultimately reduces the singlet to triplet state transition via intersystem crossing (ISC). This reduction in ISC affects the triplet state lifetime for the sonosensitizer molecule, followed by its energy transfer and yield of cytotoxic singlet oxygen. Therefore, nonradiative relaxation pathways need to be controlled while designing molecules to extend the excited state lifetime.

**Fig. 15.**
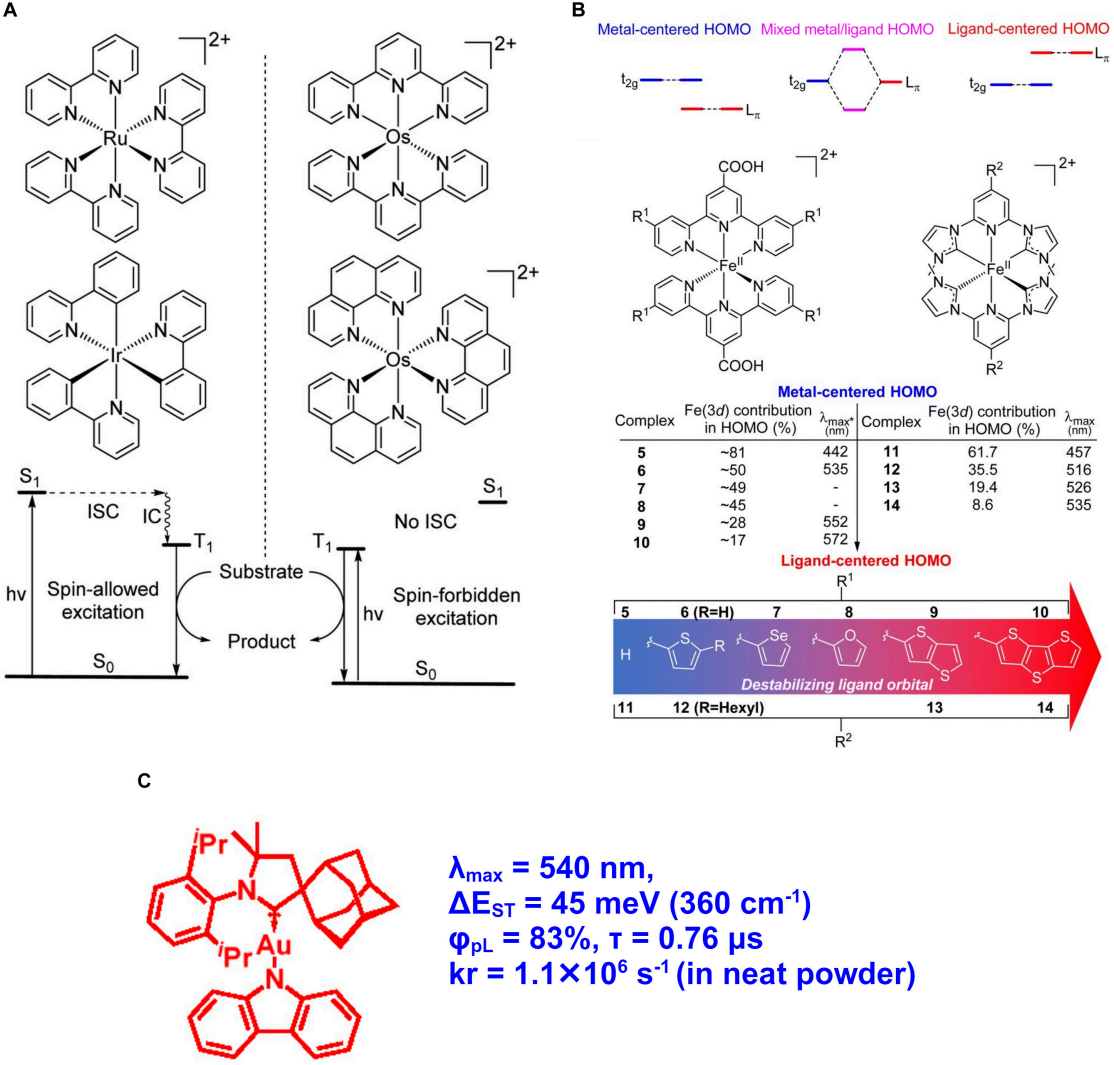
(A) The direct excitation from the ground state (S_0_) to the triplet state (T_1_) is a spin-forbidden process. However, the use of heavy metal atoms can directly excite the sensitizer molecule to its triplet state and can enhance the cytotoxic singlet oxygen generation. This figure is reproduced with permission from [101]. https://creativecommons.org/licenses/by/3.0/ (B) HOMO inversion process: Substitution of electron donor groups causing destabilizing HOMO by MC to LC HOMO state. This enhances the absorption spectrum of the sensitizer molecule. This figure is reproduced with permission from [101]. https://creativecommons.org/licenses/by/3.0/ (C) Structure of “cMa” complexes (c: carbene; M: coinage *d*^10^ metal; a: anionic amide ligand). These recently developed complexes have high potential for use as novel sonosensitizer molecules as alternatives to MC complexes due to their low cost, low toxicity, and, most importantly, the capability of avoiding nonradiative energy transfer. This figure is reproduced (adapted) with permission from [98], Copyright 2024, American Chemical Society.

Efficient spin-orbit coupling (SOC) is another criterion that is capable of producing a triplet state in sensitizers. The introduction of heavy metal atoms in molecules introduces effective SOC and accelerates ISC, producing a triplet state [98,100]. The selection of biocompatible heavy atoms will be key to making SDT pragmatic. Direct excitation of the sensitizer from its ground state to the triplet state is another possible approach to improve the triplet state lifetime and, consequently, singlet oxygen generation. This direct triplet state excitation process is a spin-forbidden process. However, heavy metals, such as osmium II complexes, with large spin-orbit-coupling constants can be excited directly to their triplet state [101] and, thereby, are expected to improve SDT efficacy (Fig. 15A).

Metal-containing porphyrin structures/metal complexes are promising alternatives to conventional porphyrin-based sensitizers. Suitable substitution by donor groups (such as furan, thiophene, and selenophene) with higher *π* conjugation can destabilize the ligand orbitals. This can lead to the shifting of metal-centered (MC) HOMO to ligand-centered (LC) HOMO, often known as “HOMO inversion” [101,102]. The HOMO inversion results in an improvement in light absorption over a higher range (Fig. 15B). Further, the LC state can participate in the singlet oxygen-independent type I reaction and, thus, have the potential to overcome tumor hypoxic conditions limiting the SDT efficacy [97]. MC complexes, however, increase nonradiative decay due to their geometrical reorganization during the shifting of electron density from the metal’s *d* orbital to the ligand’s *π*^∗^ orbital [98]. To overcome this shortcoming, “cMa” (c: carbene; M: coinage *d*^10^ metal; a: anionic amide ligand) complexes have gained interest (Fig. 15C). Avoiding MC excited states through fully filled d orbitals results in generating molecules with high tunability in absorption energies, long-excited state lifetime (hundreds of nanoseconds to microseconds), and near-unity quantum yield [98].

#### Safety and toxicity of the newly developed sonosensitizers

One of the most important aspects of designing and developing new sonosensitizer molecules in clinical use is their precise delivery at the target tissue and selective accumulation within the tumor cells without inducing cytotoxic effects in healthy cells. In addition, these molecules must be rapidly excreted from the human body [103]. The targeted delivery of the sensitizer can be accomplished by encapsulation within a vehicle (e.g., nanoparticles, mesoporous silica, and liposomes) [104]. Although inorganic and synthetic vehicles have gained attention for this purpose, they suffer from inherent toxicity and immunogenicity depending on the dosage. Exosomes, which are nanoscale vesicles (~30 to 150 nm) with lipid membranes, can deliver the sensitizers at the tumor site. These extracellular vesicles are cell specific with none or minimal immunogenicity and a long blood circulation half-life. Therefore, exosomes have the potential for clinical use in sonosensitizer delivery with a high efficacy [105].

#### Disruption of redox homeostasis, overcoming tumor hypoxia

**Fig. 16.**
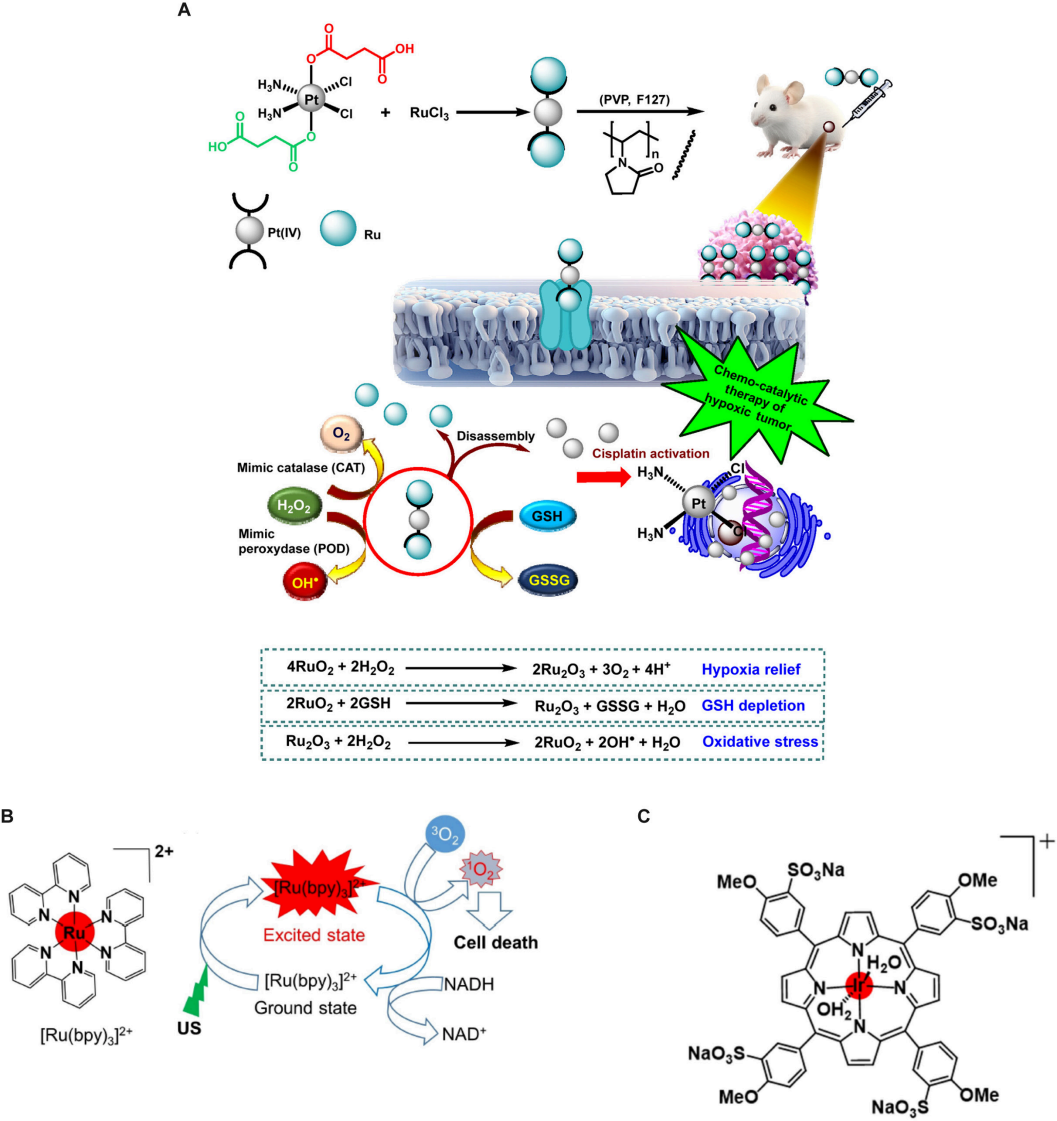
(A) Pt-Ru-based hybrid complex exhibits multienzymatic catalytic activity and hence can act as a potential sensitizer with tumor hypoxia alleviation as well as a GSH-depleting substance. This can lead to an increase in SDT efficacy. This image is reproduced from [109]. https://creativecommons.org/licenses/by-nc-nd/4.0/ (B and C) Ru (II)-based and Ir-based metal complexes. These metal complexes have a low LUMO-HOMO energy gap, oxidize NADH to NAD^+^, and thereby can play a vital role in GSH depletion and ROS yield. These images are reproduced with permission from [108], Copyright 2022 Wiley-VCH GmbH.

Nicotinamide adenine dinucleotide phosphate (NADPH) is one of the most vital electron donors that can produce GSH via glutathione disulfide (GSSG) reduction in the presence of the GSH reductase enzyme [106]. The GSH concentration in TME is higher (0.5 to 10 mM) compared to the normal cells [107]. A high GSH concentration either scavenges ROS directly or can act as a cosubstrate for GSH peroxidase (GPX), which can reduce H_2_O_2_ to H_2_O or alcohol. A high GSH level can also resist cellular apoptosis due to its binding with the antiapoptotic protein Bcl-2 in mitochondria [107] and thereby limit SDT efficacy. Further, as the TME suffers from hypoxia, it limits ROS generation and, ultimately, affects SDT efficacy. Metal-porphyrin complexes such as the Ru (II) complex and Ir (III) complex can act as potential sonosensitizers since they promote the oxidation of NADH [reduced form of nicotinamide adenine dinucleotide (oxidized form) (NAD^+^)] to NAD^+^ and deplete GSH [108]. The metal complexes also exhibit a lower LUMO-HOMO energy gap, and, therefore, low ultrasound energy is expected. The Pt-Ru-based hybrid complex can also facilitate GSH depletion and tumor hypoxia alleviation due to its multienzymatic catalytic functions (Fig. 16) [109].

### Improvement of preclinical investigations using 3D in vitro tumor models

Most preclinical in vitro SDT studies have employed 2D monolayer cultures. In contrast, the physiological conditions in a TME are complex, which a 2D cell culture fails to mimic. For example, the O_2_ gradient in the TME is higher at its periphery compared to its hypoxic core. Sensitizer accumulation within tumor cells ultimately determines SDT efficacy. In the case of a 2D model, surface adsorption of sensitizer is the primary cause of its accumulation within tumor cells. In the heterogeneous 3D TME, sensitizer accumulation is governed by diffusion. Thus, 3D tumor spheroids offer a cost-effective and scalable alternative for in vitro SDT studies to screen new sensitizers and their efficacy.

**Fig. 17.**
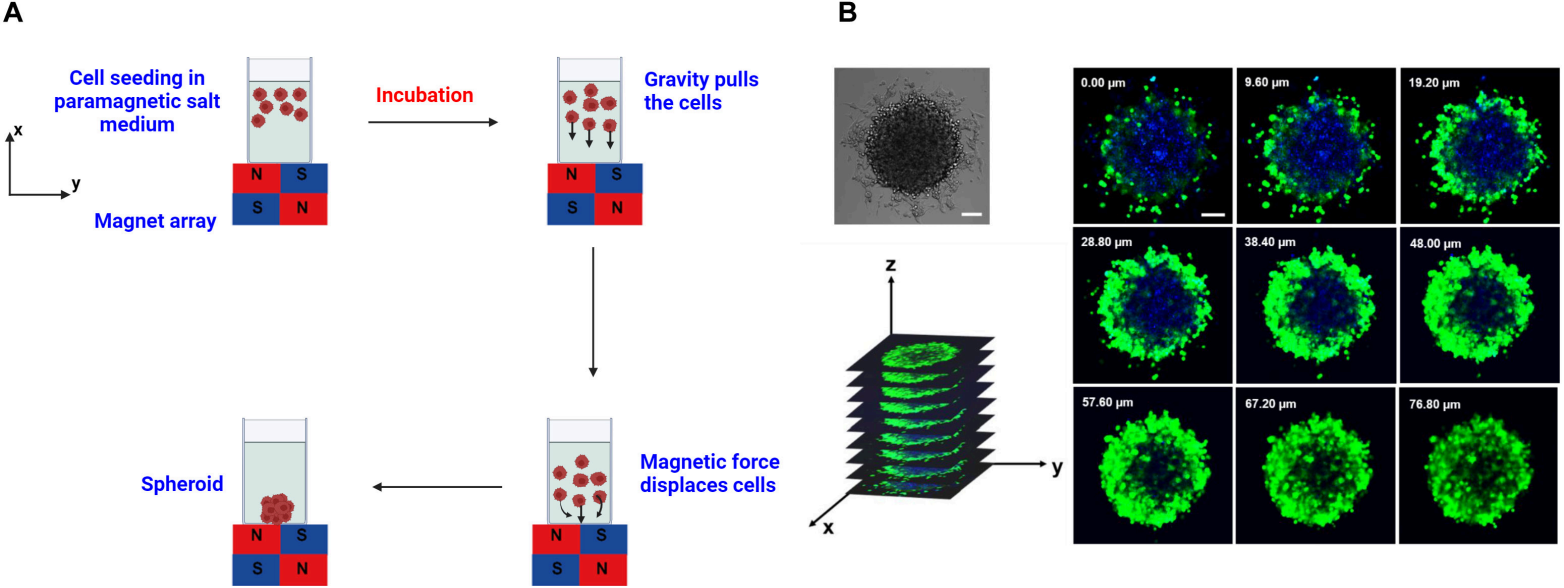
(A) Schematic representation of the magnetic field-assisted spheroid printing. Cells are seeded in a cell culture medium containing the paramagnetic agent Gadovist. Depending on the magnetic susceptibility difference between cells and their surrounding medium, a net magnetic force acts and displaces cells toward the lowest magnetic field zone. This results in the formation of cellular aggregates within 3 to 6 h depending on cell types. (B) Coculture of MCF7 and NIH 3T3 fibroblasts forms layer-by-layer cellular aggregates within 3 h. This image is reproduced with permission from [110], Copyright 2020, American Chemical Society.

Few 3D spheroid models have been used for SDT in vitro studies. Most available spheroid generation methods are tedious, lengthy, and, therefore, not scalable. We have developed a new magnetic field-guided, label-free, high-throughput spheroid printing technology (Fig. 17) [110–112]. Depending on the types of cells, our printing method generates spheroids within 3 to 6 h. This scalable technique has promising potential for use as a patient-specific sonosensitizer and drug screening platform. The feasibility of cell coculturing also makes this technology a strong platform for investigating SDT-based multimodal approaches such as SDT-CAR-T therapy.

### Ultrasound-controlled CAR-T therapy in combination with SDT

CAR-T cell-based immunotherapy is fast becoming a paradigm-shifting therapy for cancer treatment. However, solid tumor treatment using CAR-T is still in the preclinical stage, where CAR expression must be precisely controlled to avoid nonspecific targeting against normal and nonmalignant tissues (off-target tumor toxicities). Controllable CAR expression increases CAR-T cell efficacy by avoiding its exhaustion. Recently, ultrasound-based, noninvasive, remotely controlled, and inducible CAR-T therapy has shown potential for solid tumor treatment [113]. Here, multimodal therapy (SDT-ultrasound controlled CAR-T) should be targeted for future preclinical studies to increase the therapeutic efficacy with simultaneous activation of SDT and the CAR-T pathway.

### Lipid-coated oxygen microbubble-assisted SDT

Our recent theoretical investigation [114] shows that a lipid-coated O_2_ microbubble exhibits stronger sonoluminescence compared to the FDA-approved ultrasound contrast agent Lumason. It is, therefore, expected that the use of lipid-coated O_2_ microbubbles has the potential to enhance therapeutic efficacy. However, more preclinical SDT experiments are required to investigate the antitumor effect of the sonosensitizers that are currently being used in clinical trials (such as 5-ALA) in the presence of lipid-coated O_2_ microbubbles.

### Machine learning for optimizing ultrasound parameters and sonosensitizer combination

It is evident from current preclinical SDT investigations that a multimodal therapy (such as SDT-chemotherapy) can overcome the limitations of SDT when it is used alone. Therefore, focus should be given to the acoustic responses for existing FDA-approved chemotherapeutics. For example, recent investigations claimed that TMZ, which is a standard GBM treatment drug, is also responsive to the ultrasound field. Hence, the response of different drug molecules, either individually or in combinations, to ultrasound fields should be investigated. An optimum drug/sonosensitizer combination, along with the ultrasound parameters, can be obtained with machine learning techniques, which should help point toward clinical translation for different deep-tissue solid tumors using SDT.
