# Supplementary material for: Recent Advances and Future Directions in Sonodynamic Therapy for Cancer Treatment
Source: BME Front. 2024 Dec 27;2024:0080. doi: 10.34133/bmef.0080 (PMC11671681; doi:10.34133/bmef.0080)
Supplement: Supplementary 1 — Table S1 [file bmef.0080.f1.pdf]

Recent advances and future directions in sonodynamic therapy for cancer treatment

Priyankan Datta[1], Sreejesh Moolayadukkam[1,2], Dhrubajyoti Chowdhury[3], Adnan Rayes[4], Nan Sook Lee[4], Rakesh P. Sahu[5,6], Qifa Zhou[4*], Ishwar K. Puri[1,3,4*]

[1]Department of Aerospace and Mechanical Engineering, University of Southern California, Los Angeles, CA 90089, USA
[2]Iovine and Young Academy, University of Southern California, Los Angeles, CA 90089, United States
[3]Mork Family Department of Chemical Engineering and Material Science, University of Southern California, Los Angeles, CA 90089, United States
[4]Alfred E. Mann Department of Biomedical Engineering, University of Southern California, Los Angeles, CA 90089, United States
[5]Department of Materials Science and Engineering, McMaster University, Hamilton, ON L8S 4L8, Canada
[6]School of Biomedical Engineering, McMaster University, Hamilton, ON L8S 4L8, Canada

[*]Corresponding authors: qifazhou@usc.edu, ikpuri@usc.edu

Table S1a. Summary of different organic sensitizer subclasses reported in *in vivo* and *in vitro* studies

**Sensitizer type**	**Frequency range (MHz)**	**DF range (%)**	**Intensity range (Wcm^{-2}) /Total energy range(J)**	**Pulse repetition frequency range (Hz)**	**Ultrasound duration range**	**Biological model (*in-vitro*/*in-vivo*)**	**Ref.**
**A) Porphyrin-based sonosensitizer**							
5-ALA	0.22-1.06	10-100	0.16-25, 400-4000 J	100	1-20 min	Glioma, glioblastoma	[1-5]
Hematoporphyrin dihydrochloride	1.92	-	1.8	-	-	Sarcoma	[6]
Hematoporphyrin	1.2-1.92	-	0.5-3.18	-	-	Leukemia sarcoma	[7, 8]
DCPH-P-Na(I) (Porphyrin derivative)	1.0	50	0.5-2.0	-	-	Lung cancer, gastric cancer, pancreatic cancer,	[9]

						prostate cancer	
Protofrin II	2.0	-	6.0	-	-	Sarcoma	[10]
Protoporphyrin-IX	1-2.2	10	0.8-5	100	30 s -3 min	Leukemia, carcinoma, breast cancer, sarcoma,	[11-19]
Hematoporphyrin + Ga-porphyrin	2-5	-	-	-	5 min	Sarcoma, carcinoma	[20]
Gallium-porphyrin complex (ATX-70)	0.5-1.93	-	3-16	-	3-5 min	Colon cancer, osteosarcoma, leukemia	[21-23]
Haematoporphyrin monomethyl ether (HMME)	1-10.5	-	0.5-1.5	-	1-2 min	Glioma, osteosarcoma, glioma, carcinoma, leukemia	[24-28]
Haematoporphyrin monomethyl ether (HMME)/Doxorubicin	1.2	-	1.0	-	2 min	Carcinoma	[29]
Photolon	1.0	-	0.4-1	-	10 min	Glioma	[30]
DEG (7,12- bis(1-(2-(2-hydroxyethoxy)ethoxy)ethyl)-3,8,13,17-tetramethylporphyrin-2,18-dipropionatomanganese)	1.0	10	0.5-2.0	-	1-2 min	Giant cell carcinoma, adenocarcinoma, squamous cell carcinoma, tubular carcinoma, somatostatinoma, ductal adenocarcinoma, breast cancer	[31]

Sino-porphyrin	0.50-1.5	20-50	0.50	1-10	1-10 min	Glioblastoma, glioma	[32-34]
**B) Xanthene sensitizer**							
Rose bengal	1-1.93	50	0.5-20	100	3-4 min	Lymphoma, prostrate cancer, sarcoma, carcinoma, glioma	[7, 20, 35-38]
Fluorescein	0.35	-	2-6	-	-	Glioma	[39]
**C) Chlorin**							
Photochemically active chlorine,mono-l-aspartyl chlorin e6 (NPe6)	1.93	-	-	-	3 min	Leukemia	[40]
**D) Anti-inflammatory drugs**							
Methylene blue	1.7-2	-	0.24-0.46	-	30 s-5 min	Sarcoma, carcinoma	[41, 42]
Curcumin	0.86-1	-	0.5-2	-	1-15 min	Leukemia	[43, 44]
**E) Aminocradines**							
Acridine orange	-	-	2.0	-	1 min	Sarcoma	[45]
**F) Napthalelone**							
Hypocrellin	1.56	-	4.0	0.4-0.8	1-3 min	Cervical cancer	[46]
Hypocrellin B	1.7	-	0.65	-	15 s	Carcinoma	[47]
**G) Pthalocyanine**							
di-sulfo-di-phthalimidomethyl phthalolcyanine zinc (ZnPcS2P2)	1.0	-	0.5	-	2 min	Glioblastoma	[48]
**H) Quinolone**							

PEGylated LFLXs (Lomefloxacin)	2.0	-	2.0	-	30 s	Sarcoma	[49]
**I) Anti-tumor drug**							
Temozolomide	1-1.5	20-50	1-20	100	6-10 min	Glioblastoma	[50]
**J)**							
BODIPY	1.0	50	1.5		3 min	Carcinoma	[53]

Table S1b. Summary of different inorganic sensitizer subclasses reported in *in vivo* and *in vitro* studies

**A) Nanoparticle-based sonosensitizers**	**Frequency range (MHz)**	**DF range (%)**	**Intensity range (Wcm^{-2})/ Total energy range (J)**	**Pulse repetition frequency range (Hz)**	**Ultrasound duration range**	**Biological model (*in-vitro*/*in-vivo*)**	**Ref.**
Au-TiO_2	1.5	10	-	1	30 s	Carcinoma	[54]
α-Fe_2O_3/Pt	1.0	-	2.0	-	-	Breast cancer	[55]
TiO_{2-x}/Ti_3C_2	-	-	1.0	-	5 min	Breast cancer	[56]
CuO_2/Ti_3C_2	1.0	-	2.0	-	-	Glioma	[57]
TiO_2	1.0	50	0.3-1	5	10 s-2 min	Melanoma	[58]
Pt/Pd	1.0	50	1.0	-	-	Breast cancer	[59]
MnO_2/FeOOH	1.0	-	1.5	-	2 min	Breast cancer	[60]
ZnO	1.0	50	0.7-1	-	-	Breast cancer	[61]
$NiFe_2O_4$/C	1.0	-	1.0	-	1 min	Melanoma	[62]
Au	1.866	-	-	-	5 min	Epidermal carcinoma, colorectal cancer, breast adenocarcinoma	[63]
$PtCu_3$	0.035	-	3.0	-	5-10 min	Breast cancer	[64]
MoS_2	1.0	-	1.0	-	15 min	Ovarian cancer	[65]
Fe_3O_4	1.0	50	1-1.5	-	-	Breast cancer	[66]
Hydrated fullerene	1.92-2		3.0		15 min	Colon adenocarcinoma	[67]
Black Phosphorous	1.0	40	1-2	-	5 min	Breast cancer	[68]
Bismuth Ferrite	0.04-3	50	3.0	-	5 min	Cervical cancer	[69]

Graphene	1.0	-	0.8	-	30 s	Ovarian adenocarcinoma	[70]
Au/Zno/Graphene	1.0	50	1.0	-	2-4 min	Colorectal carcinoma, Breast cancer	[71]
TiN	0.04	50	3.0	-	5-10 min	Breast cancer	[72]
Black Phosphorous/Au /Polypyrrole	-	-	1.0	-	1-3 min	Breast cancer	[73]
Fe/VS_2	0.04	50	4.5-6.5	-	10 min	Breast cancer	[74]
$Cu\text{-}CuFe_2O_4$	-	-	2.0	-	900	Breast adenocarcinoma	[75]
Pt/TiO_2	1.0	50	1.5	-		Breast cancer	[76]
Nb_2C	1.0	50	1-1.5		0-5 min	Breast cancer	[77]
Ti_3C_2/TiO_2	0.04-1	40-50	1.5	-	10 min	Breast cancer	[78]
Polythiophene/ Nb_2C	1.0	-	1.5	-	10 min	Breast cancer	[79]
Si	0.003-0.88	-	0.05-1	-	-	Larynx squamous cell carcinoma	[80]
$Au\text{-}RuO_2$	1.0	50	1.5	-	5 min	Squamous cell carcinoma	[81]
Boron-based	1.0	50	1.0	-	-	Breast cancer	[82]
Polypyrrole-coated carbon nanotube	1.0	-	1.0	-	1 min	Malignant melanoma	[83]
TiO_2/C	1.0	50	1.8	-	90 s	Breast cancer	[84]
**B) Supramolecular sensitizers**							
Aggregation-induced emission luminogen	1.0	-	3.0	-	4 min	Breast cancer	[85]
Fe^{3+}/indocyanine green	1-2.86	30	1.61	-	5-10 min	Hepatoma	[86]
Fe-Hematoporphyrin	1.0	50	1.75	-	3-10 min	Colorectal carcinoma	[87]
PCN-224/Pt	3.0	-	0.1-1.5	-	5-10 min	Pancreatic cancer	[88]
Fe-MIL-88B-NH2- H4TBAP	1.0	50	1.5	-	5 min	Lung adenocarcinoma	[89]
**C) Metal-organic framework (MOF)-based sonosensitizers**							

Cyanine-rhenium(I) tricarbonyl	3.0	-	0.3	-	15 min	Breast cancer	[90]
Zn(II) Pt(II) Porphyrin Complex	1.866	-	1.5	-	5 min	Collarectal adenocarcinoma	[91]
Iridic-Porphyrin Complex	3.0	-	0.3	-	0-25 min	Breast cancer	[92]
Copper	1.0	-	1.0	-	10 min	Breast adenocarcinoma	[93]
Hf/Au	1.0	50	1.5	-	5 min	Breast cancer	[94]
Mn/Porphyrin	1.0	30-50	0.9-1	-	10 min	Hepatocellular carcinoma	[95]
Ruthernium-based polypyridinal metal complex	3	-	0-0.3	-	0-25 min	Breast cancer	[96]

References

1. Ohmura, T., et al., *Sonodynamic therapy with 5-aminolevulinic acid and focused ultrasound for deep-seated intracranial glioma in rat.* Anticancer research, 2011. **31**(7): p. 2527-2534.
2. Jeong, E.-J., et al., *Sonodynamically Induced Antitumor Effects of 5-Aminolevulinic Acid and Fractionated Ultrasound Irradiation in an Orthotopic Rat Glioma Model.* Ultrasound in medicine & biology, 2012. **38**(12): p. 2143-2150.
3. Endo, S., et al., *Porphyrin Derivatives-Mediated Sonodynamic Therapy for Malignant Gliomas In Vitro.* Ultrasound in medicine & biology, 2015. **41**(9): p. 2458-2465.
4. Wu, S.K., et al., *MR-guided Focused Ultrasound Facilitates Sonodynamic Therapy with 5-Aminolevulinic Acid in a Rat Glioma Model.* Scientific reports, 2019. **9**(1): p. 10465-10.
5. Yoshida, M., et al., *Sonodynamic Therapy for Malignant Glioma Using 220-kHz Transcranial Magnetic Resonance Imaging-Guided Focused Ultrasound and 5-Aminolevulinic acid.* Ultrasound in medicine & biology, 2019. **45**(2): p. 526-538.
6. Umemura, S.i., et al., *Mechanism of Cell Damage by Ultrasound in Combination with Hematoporphyrin.* Cancer science, 1990. **81**(9): p. 962-966.
7. Hiraoka, W., et al., *Comparison between sonodynamic effect and photodynamic effect with photosensitizers on free radical formation and cell killing.* Ultrasonics Sonochemistry, 2006. **13**(6): p. 535-542.
8. Yumita, N., et al., *Hematoporphyrin as a sensitizer of cell-damaging effect of ultrasound.* Jpn J Cancer Res, 1989. **80**(3): p. 219-22.
9. Hachimine, K., et al., *Sonodynamic therapy of cancer using a novel porphyrin derivative, DCPH-P-Na(I), which is devoid of photosensitivity.* Cancer Science, 2007. **98**(6): p. 916-920.

10. Yumita, N. and S.-i. Umemura, *Ultrasonically induced cell damage and membrane lipid peroxidation by photofrin II: mechanism of sonodynamic activation.* Journal of Medical Ultrasonics, 2004. **31**(1): p. 35-40.
11. Shanei, A., et al., *Sonodynamic Therapy Using Protoporphyrin IX Conjugated to Gold Nanoparticles: An In Vivo Study on a Colon Tumor Model.* (2008-3866 (Print)).
12. Guo, S., et al., *Apoptosis of THP-1 macrophages induced by protoporphyrin IX-mediated sonodynamic therapy.* Int J Nanomedicine, 2013. **8**: p. 2239-46.
13. Sazgarnia, A., et al., *A Novel Nanosonosensitizer for Sonodynamic Therapy.* Journal of Ultrasound in Medicine, 2011. **30**(10): p. 1321-1329.
14. Li, Y., et al., *Apoptosis induced by sonodynamic treatment by protoporphyrin IX on MDA-MB-231 cells.* Ultrasonics, 2012. **52**(4): p. 490-496.
15. Liu, Q., et al., *Comparison between sonodynamic effect with protoporphyrin IX and hematoporphyrin on sarcoma 180.* Cancer Chemother Pharmacol, 2007. **60**(5): p. 671-80.
16. Wang, X., et al., *Sonodynamically induced anti-tumor effect with protoporphyrin IX on hepatoma-22 solid tumor.* Ultrasonics, 2011. **51**(5): p. 539-546.
17. Liu, Q., et al., *Sonodynamic effects of protoporphyrin IX disodium salt on isolated sarcoma 180 cells.* Ultrasonics, 2006. **45**(1): p. 56-60.
18. Wang, X.B., et al., *Enhancement of apoptosis by sonodynamic therapy with protoporphyrin IX in isolate sarcoma 180 cells.* Cancer Biother Radiopharm, 2008. **23**(2): p. 238-46.
19. Wang, X.B., et al., *Sonodynamically induced apoptosis by protoporphyrin IX on hepatoma-22 cells in vitro.* Ultrasound Med Biol, 2010. **36**(4): p. 667-76.
20. Umemura, S., et al., *Sonodynamic approaches to tumor treatment.* International Congress Series, 2004. **1274**: p. 164-168.
21. Yumita, N., et al., *Sonodynamically induced antitumor effect of gallium-porphyrin complex by focused ultrasound on experimental kidney tumor.* Cancer Letters, 1997. **112**(1): p. 79-86.
22. Yumita, N., et al., *Sonodynamic therapy on chemically induced mammary tumor: pharmacokinetics, tissue distribution and sonodynamically induced antitumor effect of gallium–porphyrin complex ATX-70.* Cancer Chemotherapy and Pharmacology, 2007. **60**(6): p. 891-897.
23. Yumita, N., et al., *Sonodynamically induced apoptosis and active oxygen generation by gallium–porphyrin complex, ATX-70.* Cancer Chemotherapy and Pharmacology, 2010. **66**(6): p. 1071-1078.
24. Li, J.-h., et al., *In vitro study of haematoporphyrin monomethyl ether-mediated sonodynamic effects on C6 glioma cells.* Neurological sciences, 2008. **29**(4): p. 229-235.
25. Tian, Z., et al., *Hematoporphyrin Monomethyl Ether Enhances the Killing Action of Ultrasound on Osteosarcoma In Vivo.* Journal of Ultrasound in Medicine, 2009. **28**(12): p. 1695-1702.
26. Li, J.H., et al., *Calcium overload induces C6 rat glioma cell apoptosis in sonodynamic therapy.* Int J Radiat Biol, 2011. **87**(10): p. 1061-6.
27. Jin, H., et al., *Sonodynamic effects of hematoporphyrin monomethyl ether on CNE-2 cells detected by atomic force microscopy.* J Cell Biochem, 2011. **112**(1): p. 169-78.
28. Su, X., et al., *Apoptosis of U937 cells induced by hematoporphyrin monomethyl ether-mediated sonodynamic action.* Cancer Biother Radiopharm, 2013. **28**(3): p. 207-17.

29. Liang, L., et al., *The Combined Effects of Hematoporphyrin Monomethyl Ether-SDT and Doxorubicin on the Proliferation of QBC939 Cell Lines.* Ultrasound in Medicine & Biology, 2013. **39**(1): p. 146-160.
30. Tserkovsky, D.A., et al., *Effects of combined sonodynamic and photodynamic therapies with photolon on a glioma C6 tumor model.* Exp Oncol, 2012. **34**(4): p. 332-5.
31. Tsuru, H., et al., *Tumor growth inhibition by sonodynamic therapy using a novel sonosensitizer.* Free Radical Biology and Medicine, 2012. **53**(3): p. 464-472.
32. Liu, H., et al., *Theranostic nanosensitizers for highly efficient MR/fluorescence imaging-guided sonodynamic therapy of gliomas.* J Cell Mol Med, 2018. **22**(11): p. 5394-5405.
33. Pi, Z., et al., *Sonodynamic Therapy on Intracranial Glioblastoma Xenografts Using Sinoporphyrin Sodium Delivered by Ultrasound with Microbubbles.* Ann Biomed Eng, 2019. **47**(2): p. 549-562.
34. Sun, Y., et al., *Tumor targeting DVDMS-nanoliposomes for an enhanced sonodynamic therapy of gliomas.* Biomaterials Science, 2019. **7**(3): p. 985-994.
35. Nomikou, N., et al., *Microbubble–sonosensitiser conjugates as therapeutics in sonodynamic therapy.* Chemical Communications, 2012. **48**(67): p. 8332-8334.
36. Sugita, N., et al., *Sonodynamically induced cell damage using rose bengal derivative.* Anticancer Res, 2010. **30**(9): p. 3361-6.
37. Umemura, S.-i., et al., *Sonodynamically induced effect of rose bengal on isolated sarcoma 180 cells.* Cancer Chemotherapy and Pharmacology, 1999. **43**(5): p. 389-393.
38. Yoshino, S., et al., *Effects of focused ultrasound sonodynamic treatment on the rat blood-brain barrier.* Anticancer Res, 2009. **29**(3): p. 889-95.
39. Prada, F., et al., *Fluorescein-mediated sonodynamic therapy in a rat glioma model.* Journal of Neuro-Oncology, 2020. **148**(3): p. 445-454.
40. Yumita, N., et al., *Sonodynamically-induced apoptosis, necrosis, and active oxygen generation by mono-l-aspartyl chlorin e6.* Cancer Sci, 2008. **99**(1): p. 166-72.
41. Komori, C., et al., *The Sonodynamic Antitumor Effect of Methylene Blue on Sarcoma180 Cells In Vitro.* Anticancer Research, 2009. **29**(6): p. 2411.
42. Xiang, J., et al., *Apoptosis of ovarian cancer cells induced by methylene blue-mediated sonodynamic action.* Ultrasonics, 2011. **51**(3): p. 390-395.
43. Zheng, L., et al., *Apoptosis of THP-1 derived macrophages induced by sonodynamic therapy using a new sonosensitizer hydroxyl acetylated curcumin.* PLoS One, 2014. **9**(3): p. e93133.
44. Wang, F., et al., *The sonodynamic effect of curcumin on THP-1 cell-derived macrophages.* Biomed Res Int, 2013. **2013**: p. 737264.
45. Suzuki, N., et al., *Antitumor effect of acridine orange under ultrasonic irradiation in vitro.* Anticancer Res, 2007. **27**(6b): p. 4179-84.
46. Meng, Y., et al., *Water-soluble and biocompatible sono/photosensitizer nanoparticles for enhanced cancer therapy.* Nanomedicine (Lond), 2010. **5**(10): p. 1559-69.
47. Wang, P., et al., *Hypocrellin B enhances ultrasound-induced cell death of nasopharyngeal carcinoma cells.* Ultrasound Med Biol, 2010. **36**(2): p. 336-42.
48. Chen, Z., et al., *Use of a novel sonosensitizer in sonodynamic therapy of U251 glioma cells in vitro.* Exp Ther Med, 2012. **3**(2): p. 273-278.
49. Komori, C., et al., *Sonodynamic Effects of Lomefloxacin Derivatives Conjugated with Methoxy Polyethylene Glycol on Sarcoma 180 Cells.* Anticancer Research, 2009. **29**(1): p. 243.

50. Wang, F., et al., *Ultrasound-excited temozolomide sonosensitization induces necroptosis in glioblastoma.* Cancer letters, 2023. **554**: p. 216033-216033.
51. Song, S., et al., *Low-intensity pulsed ultrasound-generated singlet oxygen induces telomere damage leading to glioma stem cell awakening from quiescence.* iScience, 2022. **25**(1): p. 103558-103558.
52. Zhou, Y., et al., *Temozolomide-based sonodynamic therapy induces immunogenic cell death in glioma.* Clinical immunology (Orlando, Fla.), 2023. **256**: p. 109772-109772.
53. Li, X., et al., *Exploring BODIPY derivatives as sonosensitizers for anticancer sonodynamic therapy.* European journal of medicinal chemistry, 2024. **264**: p. 116035-116035.
54. Deepagan, V.G., et al., *Long-Circulating Au-TiO2 Nanocomposite as a Sonosensitizer for ROS-Mediated Eradication of Cancer.* Nano Letters, 2016. **16**(10): p. 6257-6264.
55. Zhang, T., et al., *α-Fe2O3@Pt heterostructure particles to enable sonodynamic therapy with self-supplied O2 and imaging-guidance.* Journal of nanobiotechnology, 2021. **19**(1): p. 1-358.
56. Zhang, D.-Y., et al., *In-situ TiO2-x decoration of titanium carbide MXene for photo/sono-responsive antitumor theranostics.* Journal of Nanobiotechnology, 2022. **20**(1): p. 53.
57. Zhang, M., et al., *Two-Dimensional MXene-Originated In Situ Nanosonosensitizer Generation for Augmented and Synergistic Sonodynamic Tumor Nanotherapy.* ACS Nano, 2022. **16**(6): p. 9938-9952.
58. Harada, Y., et al., *Ultrasound activation of TiO2 in melanoma tumors.* Journal of Controlled Release, 2011. **149**(2): p. 190-195.
59. Zhou, Z., et al., *Bimetallic PdPt-based nanocatalysts for Photothermal-Augmented tumor starvation and sonodynamic therapy in NIR-II biowindow assisted by an oxygen Self-Supply strategy.* Chemical Engineering Journal, 2022. **435**: p. 135085.
60. Liu, Q., et al., *Ultrathin-FeOOH-Coated MnO2 Sonosensitizers with Boosted Reactive Oxygen Species Yield and Remodeled Tumor Microenvironment for Efficient Cancer Therapy.* Advanced science, 2022. **9**(17): p. e2200005-n/a.
61. Liu, Y., et al., *Defect modified zinc oxide with augmenting sonodynamic reactive oxygen species generation.* Biomaterials, 2020. **251**: p. 120075.
62. Gorgizadeh, M., et al., *Sonodynamic cancer therapy by a nickel ferrite/carbon nanocomposite on melanoma tumor: In vitro and in vivo studies.* Photodiagnosis and Photodynamic Therapy, 2019. **27**: p. 27-33.
63. Brazzale, C., et al., *Enhanced selective sonosensitizing efficacy of ultrasound-based anticancer treatment by targeted gold nanoparticles.* Nanomedicine (Lond), 2016. **11**(23): p. 3053-3070.
64. Zhong, X., et al., *GSH-Depleted PtCu3 Nanocages for Chemodynamic- Enhanced Sonodynamic Cancer Therapy.* Advanced Functional Materials, 2019. **30**.
65. Ge, H., et al., *Effective treatment of cisplatin-resistant ovarian tumors with a MoS2-based sonosensitizer and nanoenzyme capable of reversing the resistant-microenvironment and enhancing ferroptosis and apoptosis.* Chemical Engineering Journal, 2022. **446**: p. 137040.
66. Wang, D., et al., *Magnetic covalent organic framework-based nanoadjuvant for multi-amplify sonodynamic antitumor therapy effect.* Nano Today, 2024. **54**: p. 102088.
67. Yumita, N., et al., *Sonodynamically-induced Anticancer Effects by Functionalized Fullerenes.* Anticancer Research, 2013. **33**(8): p. 3145.

68. Ouyang, J., et al., *Two dimensional semiconductors for ultrasound-mediated cancer therapy: the case of black phosphorus nanosheets.* Chemical Communications, 2018. **54**(23): p. 2874-2877.
69. Feng, L., et al., *Multifunctional Bismuth Ferrite Nanocatalysts with Optical and Magnetic Functions for Ultrasound-Enhanced Tumor Theranostics.* ACS Nano, 2020. **14**(6): p. 7245-7258.
70. Lee, H.R., et al., *Sonosensitizer-Functionalized Graphene Nanoribbons for Adhesion Blocking and Sonodynamic Ablation of Ovarian Cancer Spheroids.* Adv Healthc Mater, 2021. **10**(13): p. e2001368.
71. Wang, F., et al., *Integrating Au and ZnO nanoparticles onto graphene nanosheet for enhanced sonodynamic therapy.* Nano Research, 2022. **15**(10): p. 9223-9233.
72. Wang, X., et al., *Liquid exfoliation of TiN nanodots as novel sonosensitizers for photothermal-enhanced sonodynamic therapy against cancer.* Nano Today, 2021. **39**: p. 101170.
73. Chen, W., et al., *Black Phosphorus Nanosheets Integrated with Gold Nanoparticles and Polypyrrole for Synergistic Sonodynamic and Photothermal Cancer Therapy.* ACS Applied Nano Materials, 2021. **4**(8): p. 7963-7973.
74. Lei, H., et al., *Biodegradable Fe-Doped Vanadium Disulfide Theranostic Nanosheets for Enhanced Sonodynamic/Chemodynamic Therapy.* ACS Applied Materials & Interfaces, 2020. **12**(47): p. 52370-52382.
75. Gong, C., et al., *Engineering Cu-CuFe2O4 nanoenzyme for hypoxia-relief and GSH-depletion enhanced chemodynamic/sonodynamic therapy.* Chemical Engineering Journal, 2022. **435**: p. 135083.
76. Liang, S., et al., *A Novel Pt–TiO 2 Heterostructure with Oxygen-Deficient Layer as Bilaterally Enhanced Sonosensitizer for Synergistic Chemo-Sonodynamic Cancer Therapy.* Advanced Functional Materials, 2020. **30**: p. 1908598.
77. Xu, J., et al., *Self-generated Schottky barriers in niobium carbide MXene nanocatalysts for theory-oriented sonocatalytic and NIR-II photonic hyperthermia tumor therapy.* Nano Today, 2023. **48**: p. 101750.
78. Xu, J., et al., *Interfacial engineering of Ti3C2-TiO2 MXenes by managing surface oxidation behavior for enhanced sonodynamic therapy.* Acta Biomaterialia, 2024. **175**: p. 307-316.
79. Pang, E., et al., *Catalase-like pleated niobium carbide MXene loaded with polythiophene for oxygenated sonodynamic therapy in solid tumor.* Nanoscale, 2023. **15**(40): p. 16466-16471.
80. Osminkina, L.A., et al., *Silicon Nanoparticles as Amplifiers of the Ultrasonic Effect in Sonodynamic Therapy.* Bulletin of Experimental Biology and Medicine, 2016. **161**(2): p. 296-299.
81. Yin, C., et al., *Live bio-nano-sonosensitizer targets malignant tumors in synergistic therapy.* Acta Biomaterialia, 2023. **155**: p. 491-506.
82. Xiao, X., et al., *Boron-Based Nanosheets for Ultrasound-Mediated Synergistic Cancer Therapy.* Chemical Engineering Journal, 2022. **440**: p. 135812.
83. Behzadpour, N., et al., *Development of a Composite of Polypyrrole-Coated Carbon Nanotubes as a Sonosensitizer for Treatment of Melanoma Cancer Under Multi-Step Ultrasound Irradiation.* Ultrasound Med Biol, 2020. **46**(9): p. 2322-2334.
84. Yang, C.-C., et al. *Using C-doped TiO2 Nanoparticles as a Novel Sonosensitizer for Cancer Treatment*. Antioxidants, 2020. **9**, DOI: 10.3390/antiox9090880.

85. Jiang, W.A.-O., et al., *Peptide Supramolecular Assembly-Instructed In Situ Self-Aggregation for Stratified Targeting Sonodynamic Therapy Enhancement of AIE Luminogens.* (2198-3844 (Electronic)).
86. Lin, H., et al., *A single-step multi-level supramolecular system for cancer sonotheranostics.* Nanoscale Horizons, 2019. **4**(1): p. 190-195.
87. Xu, H., et al., *Biocompatible Fe-Hematoporphyrin coordination nanoplatforms with efficient sonodynamic-chemo effects on deep-seated tumors.* Biomaterials, 2020. **257**: p. 120239.
88. Bao, Y., et al., *Erythrocyte Membrane-Camouflaged PCN-224 Nanocarriers Integrated with Platinum Nanoparticles and Glucose Oxidase for Enhanced Tumor Sonodynamic Therapy and Synergistic Starvation Therapy.* ACS Applied Materials & Interfaces, 2021. **13**(21): p. 24532-24542.
89. Pu, Y., et al., *Sono-Controllable and ROS-Sensitive CRISPR-Cas9 Genome Editing for Augmented/Synergistic Ultrasound Tumor Nanotherapy.* Advanced Materials, 2021. **33**.
90. Zhu, J., et al., *An ultrasound activated cyanine-rhenium(i) complex for sonodynamic and gas synergistic therapy.* Chemical Communications, 2022. **58**(20): p. 3314-3317.
91. Giuntini, F., et al., *Insight into ultrasound-mediated reactive oxygen species generation by various metal-porphyrin complexes.* Free Radical Biology and Medicine, 2018. **121**: p. 190-201.
92. Xie, J., et al., *Water-Soluble Iridic–Porphyrin Complex for Non-invasive Sonodynamic and Sono-oxidation Therapy of Deep Tumors.* ACS Applied Materials & Interfaces, 2021. **13**(24): p. 27934-27944.
93. Zhang, K., et al., *Enhanced cancer therapy by hypoxia-responsive copper metal-organic frameworks nanosystem.* Biomaterials, 2020. **258**: p. 120278.
94. Cai, L., et al., *Piezoelectric Metal–Organic Frameworks Based Sonosensitizer for Enhanced Nanozyme Catalytic and Sonodynamic Therapies.* ACS Nano, 2023. **17**(8): p. 7901-7910.
95. Xu, Q., et al., *Manganese porphyrin-based metal-organic framework for synergistic sonodynamic therapy and ferroptosis in hypoxic tumors.* Theranostics, 2021. **11**(4): p. 1937-1952.
96. Liang, C., et al., *A highly potent ruthenium(II)-sonosensitizer and sonocatalyst for in vivo sonotherapy.* Nature communications, 2021. **12**(1): p. 5001-5001.
